# Mammalian gut metabolomes mirror microbiome composition and host phylogeny

**DOI:** 10.1038/s41396-021-01152-0

**Published:** 2021-12-13

**Authors:** Rachel Gregor, Maraike Probst, Stav Eyal, Alexander Aksenov, Goor Sasson, Igal Horovitz, Pieter C. Dorrestein, Michael M. Meijler, Itzhak Mizrahi

**Affiliations:** 1 Department of Chemistry, Ben-Gurion University of the Negev, Be’er Sheva, Israel; 2 National Institute of Biotechnology in the Negev, Ben-Gurion University of the Negev, Be’er Sheva, Israel; 3 Department of Life Sciences, Ben-Gurion University of the Negev, Be’er Sheva, Israel; 4 Collaborative Mass Spectrometry Innovation Center, Skaggs School of Pharmacy and Pharmaceutical Sciences, University of California San Diego, La Jolla, CA USA; 5 The Zoological Center Tel Aviv-Ramat Gan, Ramat Gan, Israel; 6 Center for Microbiome Innovation, University of California San Diego, La Jolla, CA USA; 7 Department of Pharmacology, School of Medicine, University of California San Diego, La Jolla, CA USA; 8 Department of Pediatrics, University of California San Diego, La Jolla, CA USA

**Keywords:** Metabolomics, Microbiome, Microbial ecology

## Abstract

In the past decade, studies on the mammalian gut microbiome have revealed that different animal species have distinct gut microbial compositions. The functional ramifications of this variation in microbial composition remain unclear: do these taxonomic differences indicate microbial adaptations to host-specific functionality, or are these diverse microbial communities essentially functionally redundant, as has been indicated by previous metagenomics studies? Here, we examine the metabolic content of mammalian gut microbiomes as a direct window into ecosystem function, using an untargeted metabolomics platform to analyze 101 fecal samples from a range of 25 exotic mammalian species in collaboration with a zoological center. We find that mammalian metabolomes are chemically diverse and strongly linked to microbiome composition, and that metabolome composition is further correlated to the phylogeny of the mammalian host. Specific metabolites enriched in different animal species included modified and degraded host and dietary compounds such as bile acids and triterpenoids, as well as fermentation products such as lactate and short-chain fatty acids. Our results suggest that differences in microbial taxonomic composition are indeed translated to host-specific metabolism, indicating that taxonomically distant microbiomes are more functionally diverse than redundant.

## Introduction

The variation between human gut microbiomes pales in comparison to the vast diversity of gut microbial communities found across mammalian species [1, 2]. Microbial diversity in mammalian microbiomes has been linked to a variety of traits related to host phylogeny, including host physiology, gut morphology, and in the majority of cases the diet, which is considered a cardinal factor in determining microbiome composition [2–6]. However, in cases of drastic historical dietary transitions, the microbiome does not always follow suit: for example, the giant panda has retained a carnivore-like microbiome more similar to other bear species than to other herbivores [5]. Overall, relationships between mammalian microbiomes tend to closely recapitulate the phylogenetic tree of their mammalian hosts, a phenomenon termed phylosymbiosis [7, 8]. Phylosymbiosis has been observed even within groups of closely related species, such as primates [9–11] and mice [12], with the notable exceptions of bats [2, 4], and ant- and termite-eating specialists such as anteaters and aardvarks [13].

However, little is known about the functional ramifications of the immense microbial taxonomic diversity across individuals and mammalian species, and whether it translates to host specificity for metabolic pathways. The most comprehensive metagenomics study of mammalian gut microbiomes to date found a large shared core of functional annotations across host species, indicating that despite drastic differences in microbial composition, these communities have similar metabolic potential [14]. Indeed, functional redundancy at the metagenome level has been found in a range of microbial environments, including host-associated environments such as the human gut microbiome [15, 16] and the rumen [17], as well as in environmental samples from the ocean [18] and soil [19]. These findings call into question basic assumptions about the relevance of taxonomic differences to ecosystem function [20, 21].

An alternate approach to understanding microbial communities is through their metabolites, which represent the downstream readout of gene expression. Metabolite secretion, uptake, and interactions with proteins and membranes shape the microbial environment and provide a medium through which microbes interact with one another and the host [22]. Therefore, metabolomics analysis can provide unique insights into microbial community function and functional redundancy [23, 24]. While the interpretation of metabolomics data remains more challenging than other omics disciplines, as the structures of metabolites span a large and diverse chemical space that cannot easily be analyzed modularly, recent advances such as molecular networking have gone a long way towards bridging this gap [25]. In this study, we examine the connection between taxonomic diversity and metabolic output in mammalian gut microbiomes by analyzing 101 fecal samples from a range of mammalian species, using an untargeted metabolomics platform together with 16S rRNA gene amplicon sequencing. We find that animals’ gut microbiomes and metabolomes are strongly correlated, especially for specialized metabolites. Our results indicate that differences in microbial taxonomic composition are indeed translated to host-specific microbial metabolism, suggesting that taxonomically distant microbiomes are not functionally redundant.

## Materials and methods

### Study design

We collected a total of 101 fecal samples from 25 mammalian species from The Zoological Center Tel Aviv-Ramat Gan, Israel (Table 1 and Supplementary Table S1). The animals were classified taxonomically on the order level (Carnivora, Artiodactyla, Perissodactyla, Proboscidea, and Primates) [26], by dietary group (carnivores, herbivores, and omnivores), and by gut morphology (foregut fermenters a.k.a. ruminants, monogastric hindgut fermenters, and monogastric simple gut morphologies). While the gut morphologies align with the mammalian phylogeny, this dataset contains two instances of dietary divergence within orders: the splitting of Carnivora into carnivores and omnivores, and the herbivorous gorillas as opposed to the other omnivorous primates species. Notably, while in this study the omnivorous Carnivora species (bears and coatis) did consume meat as well as fruit and vegetables, all the primates species consumed only fruits and vegetables (Table 1 and Supplementary Table S1). Multiple individuals per species were sampled whenever possible.

**Table 1:** Summary of animals sampled.

Order	Gut morphology	Diet type	Species	Samples	Taxonomy	Diet description
Primates	Simple gut	Omnivores	Red ruffed lemur	1	*Varecia variegata rubra*	Seasonal fruits and vegetables
			Black-and-white ruffed lemur	1	*Varecia variegata*	
			Mandrill	8	*Mandrillus sphinx*	
			Chimpanzee	10	*Pan troglodytes*	
			Gibbon	3	*Hylobates lar*	
			Capuchin	1	*Cebus olivaceus*	
		Herbivores	Gorilla	6	*Gorilla gorilla*	
Artiodactyla	Foregut	Herbivores	Sheep	5	*Ovis aries*	Alfalfa straw and herbivore pellets
			Goat	1	*Capra hircus*	
Carnivora	Simple gut	Omnivores	Black bear	2	*Ursus thibetanus*	Seasonal fruits and vegetables, kibble, twice a week chicken or fish
			Brown bear	5	*Ursus arctos*	
			Coati	7	*Nasua nasua*	Seasonal fruits and vegetables, kibble, mice
		Carnivores	African wild dog	1	*Lycaon pictus*	Chicken and red meat
			Arabian wolf	1	*Canis lupus arabs*	
			Tiger	2	*Panthera tigris*	
			Leopard	2	*Panthera pardus*	
			Lion	3	*Panthera leo*	
			Jungle cat	2	*Felis chaus*	
			Sand cat	1	*Felis margarita*	Mice
			Hyena	1	*Hyaena hyaena*	Red meat, occasional hardboiled egg or fruit
Perissodactyla	Hindgut	Herbivores	Rhinoceros	13	*Ceratotherium simum*	Oat straw and vicia straw, occasional fruit and vegetables
			Zebra	16	*Equus burchelli boehmi*	
			Donkey	1	*Equus asinus*	Alfalfa straw and herbivore pellets
Proboscidea	Hindgut	Herbivores	African elephant	3	*Loxodonta africana*	Wheat straw and herbivore pellets, occasional fruits and vegetables
			Asian elephant	5	*Elephas maximus*	

### Sample collection

Fecal samples were collected over the course of a 6-month period (September 2016–March 2017), and food samples were collected in March 2018. To ensure non-redundant sampling of individuals, animals were monitored by direct observation or by cameras, or were temporarily separated from others in a separate habitation compartment, with the exception of the zebras (Supplementary Table S1). Metadata was recorded including the diet, age, sex, and medical treatment of the individuals. The fecal samples were collected fresh, generally within 2 h post-voiding for daytime samples or up to 14 h for overnight samples, depending on the species and circumstances of collection. No systematic effect of sample collection time was observed for host species with multiple individuals sampled within different time frames (Supplementary Fig. S1). To ensure anaerobic conditions were maintained within the sample, only relatively high-moisture samples were collected. Samples were first transferred temporarily into a 50 mL tube pre-flushed with CO_2_, and kept on ice for up to 2 h until being processed on-site. Then, 10% w/v feces was dissolved in anaerobic sterile phosphate-buffered saline-10% glycerol solution with a total volume of 50 mL. The mixture was then flushed with CO_2_ via a gas line, divided into aliquots, and snap-frozen in liquid nitrogen immediately. The frozen samples were stored temporarily at −20 °C on-site, then transferred on ice in a cooler box to the laboratory and stored at −80 °C until analysis.

### Analytical procedures

Samples underwent three parallel analyses: (1) amplicon sequencing of 16S rRNA genes, to characterize the microbial community; (2) liquid chromatography tandem–mass spectrometry (LC–MS/MS), to characterize a wide range of semi-polar metabolites and lipids; and (3) gas chromatography–mass spectrometry (GC–MS) and gas chromatography-flame ionization detector (GC-FID), to characterize small, polar metabolites.

#### Amplicon sequencing analysis

Detailed protocols for the DNA extraction are provided in the Supplementary Information. In brief, prior to DNA extraction, samples were treated with a washing protocol adapted from Jami et al. [27] to separate adherent bacteria from fecal material, in order to reduce bias stemming from the low DNA extraction efficiency of particle-associated bacteria. The DNA extraction was performed as previously described by Stevenson et al. [28], with minor modifications as detailed in the Supplementary Information. Then, the V4 region of the 16S rRNA gene was amplified for the extracted DNA in each sample separately, using barcoded primers 515F 5′-GTGCCAGCMGCCGCGGTAA-3′ and 806R 5′-GGACTACHVGGGTWTCTAAT-3′ as described in Caporaso et al. [29]. The libraries were pooled and sequenced on a MiSeq platform (Illumina, San Diego, CA, USA) with 151 cycles from each end. The sequencing data was analyzed using the DADA2 pipeline v1.6.0 in R [30]. Separate sequencing runs were analyzed separately and subsequently merged. The median number of reads per sample was approx. 21,000, and rarefaction curves were calculated for the overall dataset as well as per sample, showing that these curves reached saturation (Supplementary Fig. S2). The number of amplicon sequencing variants (ASVs) detected per sample averaged approx. 200–300, depending on the host species (Supplementary Fig. S3). Taxonomy was assigned using the SILVA database v132 [31]. Predicted functional profiles were generated using PICRUSt2 v2.1.4, using the default settings [32].

#### In vitro digestion of dietary samples

Food samples from the animals’ diets were collected and analyzed in order to identify dietary metabolites. Samples were collected from 34 foods on-site in consultation with the zoological center’s animal nutritionist. Samples were crushed in a mortar and pestle, and 0.5 g was weighed into a 15 mL tube and frozen at −20 °C. Prior to analysis, in order to simulate initial host digestion of the dietary samples, a 2-stage in vitro digestion protocol was performed based on Gawlik-Dziki et al. [33]. In brief, samples were treated with 5 mL of an acidic pepsin solution and shaken for 2 h at 37 °C, followed by pH adjustment to approx. 6, the addition of 5 mL of simulated intestinal juice containing pancreatin and bile salts and 5 mL of potassium and sodium chloride solution, pH adjustment to approx. 7, and shaking for an additional 60 min at 37 °C. Samples were stored at −20 °C until analysis.

#### Metabolomics analysis

Detailed protocols and instrument settings for all metabolomics methods are provided in the Supplementary Information. In brief, for the LC–MS/MS analysis, samples were extracted overnight in 50% methanol using a protocol modified from Melnik et al. [34]. Samples were analyzed on an Acquity UPLC I-Class System (Waters Corporation, Milford, MA, USA) coupled to a qExactive hybrid quadrupole-Orbitrap mass spectrometer (Thermo Fisher Scientific, Waltham, MA, USA). For the GC–MS analysis, samples were extracted in a modified Bligh–Dyer procedure in a two-phase methyl-*tert*-butyl ether (MTBE), methanol, and water protocol (2:1:1) adapted from Giavalisco et al. [35]. The aqueous phase was dried under reduced pressure and stored at −80 °C. Immediately prior to analysis, a two-step derivatization process (methoximation and trimethylsilylation) was carried out according to the protocol described by Lisec et al. [36]. Samples were analyzed based on Hochberg et al. [37] on a GC-7820A instrument coupled to an MSD-5977B single-quadrupole mass spectrometer (Agilent Technologies, Santa Clara, CA, USA). For the short-chain fatty acid (SCFA) quantification, samples were extracted based on a protocol modified from Shabat et al., in which samples were acidified with metaphosphoric acid and then extracted using MTBE [38]. Samples were analyzed using a GC-7890B coupled to a flame ionization (FID) detector (Agilent Technologies), in order to detect and quantify a targeted panel of six SCFA based on a collection of standards (acetate, propionate, butyrate, valerate, isobutyrate, and isovalerate).

### Molecular networking

The metabolomics data was analyzed using the Global Natural Product Social Molecular Networking (GNPS) platform [25]. For the LC–MS/MS data, data files were converted to open format mzXML files using the GNPS batch converter, and then processed with MZmine2 v2.34 [39] based on the feature-based molecular networking workflow tutorial (batch converter and tutorial are available at: gnps.ucsd.edu). This resulted in a list of approx. 20,000 peak features, defined here as a given mass over charge ratio (m/z) eluting in a chromatographic peak at a given retention time (details regarding the MZmine2 workflow are available in the Supporting Information). These peak features were aligned across samples and normalized by the internal standard (ampicillin), and log-transformed. The peak list was subsequently filtered to 10,029 fecal peak features after removing the background and dietary signals (Supplementary Fig. S4), with a median of 1151 peak features detected per sample (Supplementary Fig. S3). The limit of detection for the peak features depends on both the instrument sensitivity and the cut-offs set in the automated analysis pipeline, and will be unique to each metabolite depending on the ease of ionization as well as possible matrix effects from the sample. A molecular network based on spectral similarity was created with the feature-based molecular networking workflow on the GNPS website, version 1.2.3 (http://gnps.ucsd.edu) [40]. Two additional tools were used to further annotate the molecular network: Network Annotation Propagation (NAP) [41], which combines annotations with *in-silico* fragmentation predictions, and MolNetEnhancer [42], which assigns chemical families to subnetworks using ClassyFire [43] classifications.

For the GC–MS data, the data files were converted to mzML format using MSconvert Proteowizard [44, 45]. The spectral deconvolution was performed using the MSHub GNPS workflow, and then the resulting list of peak features was analyzed by GC–MS molecular networking [46]. Spectral matching was performed in the GNPS workflow against public GNPS and commercial NIST and Wiley libraries. Both workflows were version 14, and the default settings were used. Post analysis, peak features were filtered by balance score, a measure of the reproducibility of fragmentation patterns across all samples, and peak features with a score lower than 50% were discarded. Additionally, peak areas in each sample were normalized by the area of the internal standard ribitol, with a noise cut-off level set as 0.01 normalized abundance, and lastly, log-transformed. A total of 347 fecal peak features passed the balance score threshold, with a median of 131 peak features detected per sample (Supplementary Fig. S3). Approximately 90% of the total peak features were present in both food and fecal samples (Supplementary Fig. S4). However, since nutrients of dietary origin such as amino acids and sugars are absorbed by the host in the small intestine [47, 48], when these molecules are present in the feces, they are less likely to be of dietary origin. Therefore, these peak features were not removed from the analysis.

### Statistical analysis

All statistical analyses were done using R v3.4.3 (R Core Team, 2017), using the packages *phyloseq* v1.27.2 [49] and *vegan* v2.5.4 [50]. Plots were created using the *ggplot2* package v3.1.0 [51]. Proportional Venn diagrams were created using the web application BioVenn [52]. In all analyses, the cutoff for significance was below a *p*-value of 0.05 after multiple hypothesis correction.

### PERMANOVA analysis

The Adonis implementation of non-parametric permutational multivariate analysis of variance (PERMANOVA) [53] was used for comparison between groups. To examine the effect of evenness of sample numbers across the data set, we performed PERMANOVA on smaller random subsets of samples per species in addition to the full datasets. To do so, we randomly subset the samples per species so that all species had *n* samples, from *n* = 1 to *n* = 5; for species with fewer than *n* individuals sampled, all samples were included. In general, PERMANOVA tests operate under the assumption of homogeneity of dispersions among groups, and in this dataset, there were significant differences between the dispersions among groups. In order to examine the effect of the sample size and evenness, we tested the homogeneities of the random *n* = 1–5 subsets and found that in all grouping categories we could find examples for which the groups were now homogenous, and the PERMANOVA tests still resulted in significant differences between the groups. In this scenario, in which the dispersal increases with increasing numbers of samples in a group, PERMANOVA tends to become more conservative (if a larger group is also more widely dispersed, a small, tightly clustered group will be more likely to fall within it, so differences in the centroids are more difficult to detect) [54]. Based on this analysis, the PERMANOVA results seem to be an accurate reflection of the data, especially since the results are evident visually in the ordination analysis.

### Hierarchical clustering analysis

Trees were created using the hclust function and analyzed using the packages *dendextend* v1.9.0 [55] and *ape* v5.2 [56] (Bray–Curtis dissimilarity and Ward’s hierarchical clustering method), and the host phylogeny tree created using the TimeTree database [57, 58]. Correlations between trees were calculated using *dendextend*’s implementations of the Pearson correlation and Baker’s Gamma Index [59]. In order to calculate the statistical significance of the Baker’s Gamma Index, a null model was tested using *dendextend* in which the labels of one tree were shuffled and the index recalculated (*n* = 999), and a *p*-value was calculated to compare the distribution of the index for the null model to the results.

### Identification of differential peak features

In order to create a shortlist of candidate differential peak features, a principal components analysis (PCA) using Euclidean distances was performed on the peak feature abundance tables for the LC–MS/MS and GC–MS data (Supplementary Fig. S5), and the top and bottom percentiles of loadings for PC1 and PC2 were extracted in order to determine the metabolite features which contributed most to the separation along these axes (Supplementary Fig. S6). These candidate lists were tested for significant associations with the host species, mammalian order, diet, and gut morphology using an indicator species analysis [60] (*indicspecies* v1.7.9 package in R, 99999 permutations). The resulting lists of metabolites and *p* values were adjusted for multiple comparisons using the Bonferroni correction, and the adjusted *p* values were filtered using a significance cutoff of 0.05. For the LC–MS/MS data, the top 5% of loadings included over 1500 peak features, and so the list was further filtered to the top 1% of loadings (319 peak features), of which 230 were significantly associated with mammalian order, diet, or gut morphology (Supplementary Table S2). Next, we used the GNPS platform to assign putative structures based on spectral matching (a level 3 annotation based on the Metabolomics Standards Initiative [61]), and 74 peak features could be assigned metabolite annotations and classified into chemical families [43]. Out of the 74 annotated significantly enriched LC–MS/MS metabolites in this study, 15 were automatically annotated via GNPS, with cosine scores ranging from 0.71–0.97; the rest were annotated manually or only at the family level based on neighboring nodes, and all annotations were manually examined. For the GC–MS data, the top 5% of loadings included 55 peak features, 25 of which were significantly associated with mammalian order, diet, or gut morphology (Supplementary Table S2). An additional 7 features were manually removed as suspected artifacts due to the sample storage in 10% glycerol, based on their similar identical distribution patterns across samples and annotations as glycerol-related compounds, resulting in a final list of 18 differential peak features, 14 of which could be annotated. The annotations in this study were not conclusively verified, for example with a commercial standard, and are therefore considered level 3 annotations based on the Metabolomics Standards Initiative [61].

## Results

### Mammalian metabolomes mirror microbiomes and demonstrate a strong phylosymbiotic signal

**Fig. 1:**
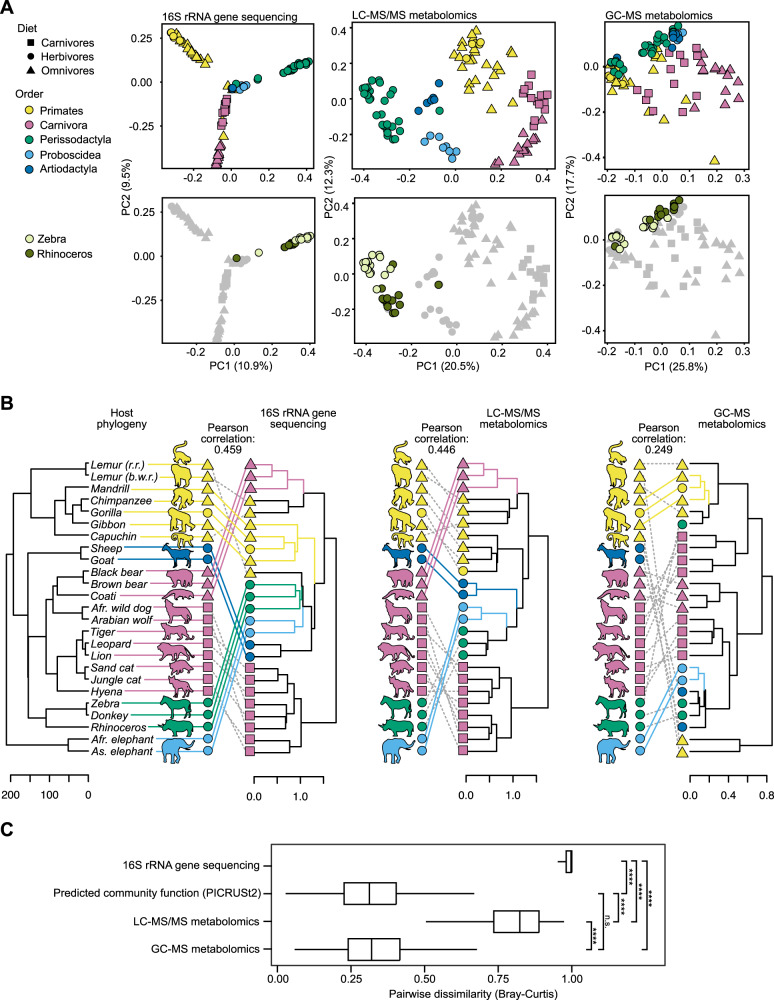
Mammalian metabolomes mirror microbiomes, reflecting functional richness across host species. **A** Top, Principal coordinates analysis (PCoA) of samples based on Bray–Curtis dissimilarity for the microbial composition (16S rRNA gene amplicon sequencing), LC–MS/MS metabolome and GC–MS metabolome. Bottom, PCoA analysis with zebras and rhinoceroses highlighted. **B** Comparison of the phylogenetic tree of the mammalian host species to the hierarchical clustering tree of samples for each data type, based on Bray–Curtis dissimilarity. Solid, colored lines connect subtrees that match in both trees. **C** Comparison of the degree of dissimilarity between samples in different data types using Bray–Curtis dissimilarity. For the boxplots, the lower and upper hinges correspond to the first and third quartiles, and outliers are not shown. The significance was determined using one-way ANOVA analysis followed by post-hoc Tukey’s test, resulting in adjusted *p* values noted as follows: *p* > 0.05 = n.s., *p* < 0.0001 = ****.

Under an assumption of functionally redundant microbial communities, we would expect that the metabolomes would be similar across fecal samples despite their diverse taxonomies. However, this hypothesis was rejected, as we found the opposite: mammalian metabolomes closely mirrored microbiome composition (Fig. 1A, top). The microbial community beta diversity was compared across samples using a principal coordinate analysis (PCoA), in order to compare the dissimilarity of the microbial composition based on Bray–Curtis dissimilarity (additional metrics showed similar trends, Supplementary Fig. S5). The microbial composition was found to be highly correlated to the liquid chromatography tandem mass spectrometry (LC–MS/MS) metabolome, as measured by a Mantel test using a Pearson correlation (*r* = 0.67, *p* = 0.0001) as well as by comparing the hierarchical clustering of all samples using a tanglegram (Pearson correlation = 0.66) (Supplementary Fig. S7). The gas chromatography mass spectrometry (GC–MS) metabolome was less correlated with both the 16S rRNA gene amplicon sequencing data (*r* = 0.27, *p* = 0.0001; tanglegram correlation = 0.21) and the LC–MS/MS data (*r* = 0.56, *p* = 0.0001; tanglegram correlation = 0.26) (Supplementary Fig. S7). Differences between these two methods of analysis (LC–MS/MS and GC–MS) are expected, as they target different classes of metabolites. The Mantel tests yield similar results when repeated with five random subsets of the samples with fewer maximum representatives per species (*n* = 1, *n* ≤ 2–5), as well as a median sample per species (Supplementary Table S3). For the LC–MS/MS to 16S rRNA gene amplicon sequencing data comparisons, all subsets were highly correlated and significant (*r* = 0.57–0.65, *p* = 0.0001), as well as for the LC–MS/MS to GC–MS subset comparisons (*r* = 0.42–0.59, *p* = 0.0001), while for the 16S rRNA gene amplicon sequencing data to GC–MS subset comparisons were significantly correlated only for the *n* ≤ 2–5 cases (*r* = 0.15–19, *p* = 0.001–0.0001). The Mantel correlations between different data types were in general not significant for intraspecies comparisons, as calculated for species for which ten or more individuals were sampled (Supplementary Table S3).

Next, we asked whether the metabolomes were associated with host traits such as phylogeny and diet, as has been shown for the microbiome. We performed a PERMANOVA analysis and found that for all three datasets, the trait that explained the most variance was the mammalian host species (*R*^2^ = 0.24–0.27, *p* = 0.001) (Table 2). Less variance was explained by dietary group (*R*^2^ = 0.14–0.20, *p* = 0.001) and by the mammalian order (*R*^2^ = 0.15–0.16, *p* = 0.001), and slightly less by the gut morphology (*R*^2^ = 0.05–0.11, *p* = 0.001). Additional factors, such as the time elapsed since sample collection (Table 2) and sex (Supplementary Table S4), explained very little variance and were not significant in most datasets. Additional possible sources of variance such as intraspecies variation accounted for less than half of the overall variance, as measured by the residuals (*R*^2^ = 0.277–0.388). In order to ensure that there was not an outsize effect from the species with many individuals sampled, we repeated the PERMANOVA analysis on five random subsets of the samples with fewer maximum representatives per species (*n* = 1, *n* ≤ 2–5) (Supplementary Table S5). In general, while the *n* = 1 and median cases mostly lost significance, for *n* ≤ 2–5 the data clustered significantly for all factors in the 16S rRNA gene amplicon sequencing data and LC–MS/MS subsets, and for some of the factors in the GC–MS subsets (Supplementary Table S5). In addition to the PERMANOVA analysis, in some cases, a clustering by host species within each mammalian order was also evident on the PCoAs (Supplementary Fig. S8). Notably, there was a clear difference between the rhinoceroses and zebras for both the microbiome and the LC–MS/MS metabolome (Fig. 1A, bottom), despite the fact that these two species were housed and fed together in the same enclosure in this study. Altogether, these results indicate that the mammalian host species is the dominant factor that explains the dissimilarity between samples.

**Table 2:** PERMANOVA (Adonis) results based on Bray–Curtis dissimilarity. Samples *n* = 101. Permutations *n* = 999. Abbreviations: *Df.* Degrees of freedom, *Sqs.* Squares.

	Df.	Sums of Sqs.	Mean Sqs.	PseudoF	*R*^2^	*p* value
**16S rRNA gene amplicon sequencing**						
Diet	2	5.88	2.94	12.61	0.13	0.001***
Gut morphology	2	4.49	2.24	9.62	0.10	0.001***
Mammalian order	2	5.53	2.77	11.86	0.12	0.001***
Host species	18	12.46	0.69	2.97	0.27	0.001***
Collection time	1	0.24	0.24	1.01	0.01	0.42
Residuals	75	17.49	0.23		0.38	
Total	100	46.09			1	
**LC–MS/MS**						
Diet	2	7.04	3.52	30.01	0.22	0.001***
Gut morphology	2	3.57	1.78	15.21	0.11	0.001***
Mammalian order	2	4.61	2.30	19.64	0.14	0.001***
Host species	18	7.49	0.42	3.55	0.24	0.001***
Collection time	1	0.29	0.29	2.46	0.01	0.005**
Residuals	75	8.80	0.12		0.28	
Total	100	31.78			1	
**GC–MS**						
Diet	2	1.00	0.50	15.11	0.16	0.001***
Gut morphology	2	0.25	0.12	3.78	0.04	0.001***
Mammalian order	2	0.97	0.49	14.75	0.15	0.001***
Host species	18	1.64	0.09	2.77	0.26	0.001***
Collection time	1	0.04	0.04	1.07	0.01	0.36
Residuals	75	2.47	0.03		0.39	
Total	100	6.36			1	

These results led us to explore the relationships between the gut microbial communities and metabolomes to the host phylogeny and to measure the degree of phylosymbiosis in this dataset [7]. To this end, we compared the topology of the microbiome and metabolome hierarchical clustering trees to the host phylogenetic tree (Fig. 1B). For species with multiple individuals sampled, a representative sample was calculated using the median values for each feature. The microbial composition tree showed the highest correlation to the host phylogeny (Pearson correlation = 0.459, Baker’s Gamma Index = 0.204, and *p* = 0.009 vs null model), closely followed by the LC–MS/MS metabolomics data (Pearson correlation = 0.446, Baker’s Gamma Index = 0.201, and *p* = 0.01), and lastly by the GC–MS metabolomics data (Pearson correlation = 0.249, Baker’s Gamma Index = 0.186, and *p* = 0.047). A similar pattern was observed in a matrix-based comparison of the host patristic distances to each dataset: the 16S rRNA gene amplicon sequencing data was the most correlated, followed by the LC–MS/MS data, while the correlation to the GC–MS data was not significant (Supplementary Table S6). These results indicate that in addition to the phylosymbiosis previously reported between the host phylogeny and the gut microbiome, there is a strong degree of agreement between the host phylogeny and the gut metabolome.

We next asked how the microbial and metabolic diversity of the samples compared to predicted community functional profiles based on gene annotations. In order to quantify how dissimilar samples were from one another for each type of data, we compared the distributions of the Bray–Curtis pairwise comparisons (Fig. 1C). The microbial composition was extremely dissimilar across samples (median dissimilarity = 0.997). However, this diversity was lost in the predicted functional profiles, created using PICRUSt2 [32], which were much more similar to one another (median dissimilarity = 0.312). The two types of metabolomics data followed a similar pattern: like the microbial communities, the LC–MS/MS metabolomes were highly diverse (median dissimilarity = 0.823), while the GC–MS metabolomes were more similar to one another (median dissimilarity = 0.321). This difference was much greater than the corresponding differences in the food sample data or technical replicates, suggesting that this effect is not due to dietary metabolites or differences in the methods of analysis (Supplementary Fig. S9), and a similar pattern was observed for intraspecies distances (Supplementary Fig. S10). Taken together, these results indicate that differences in microbiome composition are correlated with differences in metabolic output, providing evidence for functional diversity as opposed to functional redundancy.

### Differences in LC–MS/MS metabolomes are driven by diverse metabolite classes

**Fig. 2:**
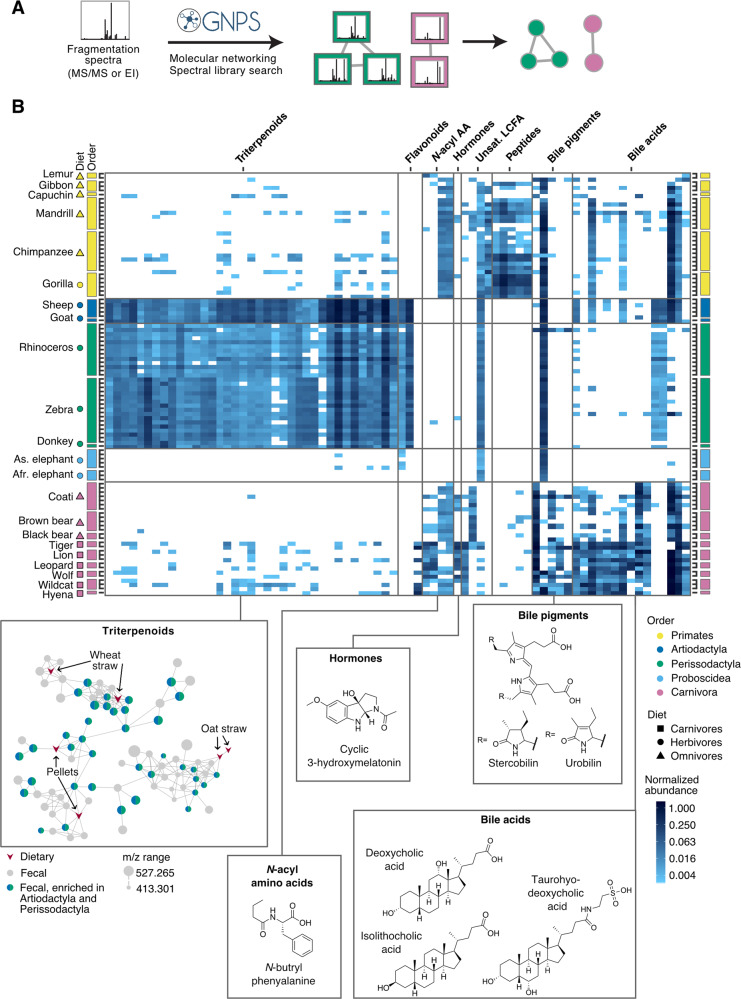
Differences in LC–MS/MS metabolomes are driven by diverse metabolite classes. **A** Molecular networking workflow to classify and putatively identify metabolites. Fragmentation patterns based on MS/MS in the LC–MS/MS dataset or electron ionization (EI) in the GC–MS dataset are organized based on spectral similarity scores. Spectra are also compared to library datasets for identification. Compounds are represented by nodes in a network, with edges connecting nodes that pass a certain spectral similarity score threshold. **B** Out of 230 metabolites significantly associated with a host characteristic, 74 could be putatively identified and classified into eight chemical families. Heatmap shows normalized abundance of peak features, with white corresponding to not detected. Examples of structures for different classes are shown on the bottom right. (Unsat. LCFA = unsaturated long-chain fatty acids; N-acyl AA = N-acyl amino acids). Bottom left, all of the herbivore-enriched triterpenoids belong to one molecular network, putatively identified as tetracyclic triterpenoids. Six plant dietary compounds showed high spectral similarity to the fecal compounds (brown arrowheads).

After observing that host phylogeny is correlated to metabolome composition, we next asked which specific metabolites are driving these differences. In order to find differential peak features likely to contribute to differences between animal groups, we performed a principal components analysis (PCA) and extracted the top percentiles of the loadings, which contributed the most to the separation on the first two PCs (Supplementary Fig. S6). These features were tested for significant associations with the host species, mammalian orders, diets, and gut morphology using an indicator species analysis [60], adjusting the *p* values for multiple comparisons using the Bonferroni correction. Next, we used the GNPS platform to assign putative structures based on spectral matching, which are level 3 annotations based on the Metabolomics Standards Initiative [61] (Fig. 2A, Supplementary Fig. S11). Out of 230 significantly differential peak features detected in the LC–MS/MS data, 74 could be assigned metabolite annotations and classified into chemical families [43] (Fig. 2B, Supplementary Fig. S12, and Supplementary Table S2). Some of the differential metabolites could be linked to the interface between the host and the microbial community, such as microbial modifications of dietary or host compounds. However, it is difficult to connect these metabolites to specific gut microbial pathways, as many of the specific enzymes and modifications involved remain unknown and unannotated.

Almost half of the differential metabolites found in the LC–MS/MS analysis belonged to one group of closely related triterpenoids and were significantly enriched in the Artiodactyla (sheep and goat) and Perissodactyla orders (zebra, rhinoceros and donkey). These 37 compounds, classified as tetracyclic triterpenoids, had very similar MS/MS spectra and clustered in a single molecular network, indicating that they are all closely structurally related (Fig. 2B), with structural variations corresponding to gains and losses of double bonds, oxygen, and hydroxyl groups, as well as desulfation (Supplementary Table S7). Six dietary compounds found in the food sample analysis also mapped to this molecular network (Fig. 2B), all of which were detected in components of these species’ diets such as straw and pellets and some of which appear to be depleted in feces (Supplementary Fig. S13). Interestingly, these compounds did not appear in the elephants, even though they also consume herbivore pellets and wheat straw. Altogether, these results suggest that these triterpenoid compounds were likely the result of microbial modification of plant dietary compounds that are specific to digestive strategies and hosts.

The bile acid class showed wide variation in its distribution across host species, with a strong dietary and phylogenetic signal (Fig. 2B). Bile salts are host-synthesized sterol-based acids, often found coupled to glycine or taurine, that aid in digestion by solubilizing lipids as well as perform important roles in host signaling. By the time bile acids reach the colon, nearly 100% have been modified by gut microbiota *via* established mechanisms of dehydrogenation, dehydroxylation, and amino acid cleavage [63, 64], as well as a recently reported novel amino acid conjugation reaction [65]. Eleven of the fifteen bile acid analogues putatively identified in this dataset were enriched in carnivores, including three known microbially modified analogues (Fig. 2B), while others were enriched in other groups including the Primates and Artiodactyla orders. The structural variation between analogues is likely due to microbial modifications, as well as the diversification of host synthesis pathways over the course of mammalian evolution [66, 67]. Indeed, none of the significantly differential bile acids were enriched in the evolutionarily distant Proboscidea order of elephants, which are reported to primarily produce C-27 bile alcohols [68].

### GC metabolomics data highlights differences in microbial degradation pathways

For the GC–MS metabolomics data, the differential metabolites covered a range of polar metabolites, including a number of known microbial degradation products as described below. Out of 18 candidate differential peak features based on the PCA loadings (Supplementary Fig. S6), we were able to putatively annotate or classify 14 using GNPS for GC–MS [46] (Supplementary Table S2). Additionally, we performed a targeted analysis of six short-chain fatty acids (SCFA) commonly found in the gut environment, using GC-FID (gas chromatography-flame ionization detector). In contrast to the LC–MS/MS analysis, here differences between animal species were less stark, with most of the differential metabolites shared across most samples with differences in abundance or ratios (Supplementary Fig. S14). Since primary metabolic pathways are much better documented than the specialized metabolism, most of the differential metabolites in the GC–MS dataset could be linked to known microbial enzymes or pathways.

**Fig. 3:**
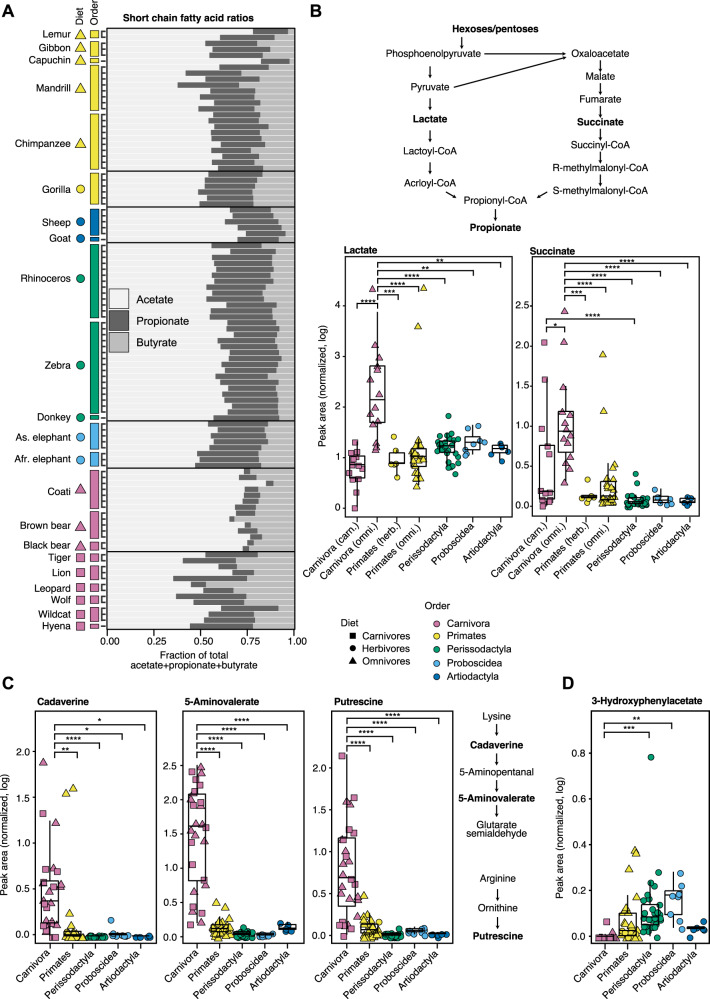
GC–MS metabolomics data highlights differences in microbial degradation pathways. **A** Different animal groups exhibited differing ratios of acetate, propionate, and butyrate. **B** Top, formation of propionate *via* succinate or lactate. Bottom, lactate and succinate levels were significantly higher in the omnivorous members of Carnivora as compared to the other groups, as measured by GC–MS. **C** Three biogenic amines were found to be significantly enriched in Carnivora. **D** 3-Hydroxyphenylacetate was significantly enriched in Perissodactyla and Proboscidea as compared to Carnivora. In all panels, all data points are shown, overlaid on a boxplot with the lower and upper hinges corresponding to the first and third quartiles. The significance was determined using one-way ANOVA analysis followed by post-hoc Tukey’s test, resulting in adjusted *p* values noted as follows: *p* < 0.05 = *, *p* < 0.01 = **, *p* < 0.001 = ***, *p* < 0.0001 = ****.

An important indication of microbial fermentation activity is the average molar ratio of acetate to propionate to butyrate, typically cited as ranging from 75:15:10 to 40:40:20 [69]. The breakdown of dietary precursors such as sugars leads to the formation of SCFA, the final output products of many microbial metabolic processes. The absolute concentration of SCFA is tightly linked to gut morphology, with faster gut transit times such as in the simple gut systems and especially Carnivora leading to less absorption and therefore higher fecal levels [70], as is seen for most of the SCFA measured here (Supplementary Fig. S15). Here, while the proportions of acetate to propionate to butyrate in the samples were in general close to the reported range, the omnivorous Carnivora species (bears and coatis) exhibited strikingly lower relative propionate levels (~73:7:21) (Fig. 3A, Supplementary Fig. S15, Supplementary Table S8). Propionate can be synthesized through three distinct microbial pathways in the gut, *via* the precursors succinate, propane-1,2-diol, or lactate [71]. Indeed, two of these precursors, lactate and succinate, exhibit elevated levels in the omnivorous Carnivora (Fig. 3B). Previous work in raccoons [72] and bears [73, 74] noted the presence of fecal lactate as well, and it was suggested that fecal lactate is characteristic of the order Carnivora due to their fast gut transit time, resulting in metabolites being excreted too quickly to be absorbed by the host [74]. However, here the levels of both lactate and succinate were higher in the omnivorous Carnivora than in carnivorous Carnivora, despite their similar gut morphologies and the even faster gut transit times in carnivores. This indicates that the elevated lactate levels are more likely a function of the composition of the microbial community than of gut morphology. Together, the low propionate levels and elevated levels of lactate and succinate suggest that the omnivorous Carnivora gut microbial communities may be less enriched in propionate-forming pathways. The gene annotations of the microbial data further strengthen this hypothesis, as propionate CoA-transferase, the last enzyme in these pathways, based on the Kyoto Encyclopedia of Genes and Genomes (KEGG) database [75], is predicted to have lower levels in the omnivorous Carnivora microbiome (Supplementary Fig. S16).

The microbial decarboxylation of nitrogen-rich amino acids results in the production of a number of key biogenic amines, including putrescine, cadaverine, and 5-aminovalerate, all of which were elevated in this dataset in the Carnivora order (Fig. 3C, Supplementary Fig. S17). Since dietary biogenic amines are quickly absorbed by the host in the small intestine [76, 77], biogenic amines found in the feces are generally attributed to gut microbial activity [78]. The synthesis of these compounds has been shown to involve multiple biosynthetic pathways shared across different microbes that exchange intermediates and products between them [79]. Cadaverine, a product of lysine degradation, is further converted to 5-aminovalerate (Fig. 3C), while putrescine is formed from arginine or ornithine degradation. When these substrates were mapped onto KEGG pathways, we found that the omnivore microbiomes were predicted to contain significantly higher levels of three key enzymes belonging to the lysine degradation pathway, as well as two enzymes in the putrescine formation pathway via ornithine (Supplementary Fig. S17). Since Carnivora consume protein-rich diets, it is likely that their microbiomes are especially adapted to utilizing these amino acid substrates, as proposed by a previous mammalian gut metagenomics study [14].

In the Perissodactyla and Proboscidea orders, 3-hydroxyphenylacetate, a degradation product of plant polyphenolic flavonoids, was enriched compared to the Carnivora (Fig. 3D). This metabolite is the primary microbial degradation product of quercetin (de-glycosolated rutin) [80–82] and proanthocyanidins [83]. This degradation process has been demonstrated in vitro by the incubation of the parent compounds with fecal slurries [84], and a handful of rumen and fecal isolate strains have been shown to degrade quercetin into 3, 4-dihydroxyphenylacetate, which can then be dehydroxylated to form 3-hydroxyphenylacetate [85, 86]. Recently, a gut dopamine dehydroxylase has been reported in *Eggerthella lenta* [87], and a follow-up study characterizing related catechol dehydroxylases showed that the conversion to 3-hydroxyphenylacetate is performed by a number of strains, including *E. lenta* and two *Gordonibacter* strains [88]. As shown above, in the LC–MS/MS metabolomics analysis, two classes of plant-derived compounds, triterpenoids and flavonoids, were enriched in the Perissodactyla and Artiodactyla (Fig. 2B). The presence of 3-hydroxyphenylacetate in the GC–MS metabolomics data is an indication that these herbivorous microbiomes may indeed be able to degrade these plant-derived metabolites.

## Discussion

A better understanding of the diversity of microbes inhabiting the mammalian gut will address fundamental questions regarding the coexistence between mammals and their microbes [89]. The question of functional redundancy is especially central: it is clear that mammalian microbiomes are diverse and that they distinctively correspond to their mammalian hosts, but do these taxonomic differences suggest host-specific functionality? Here, we examined the metabolic content of mammalian gut microbiomes as a direct window into ecosystem function, using a dual metabolomics platform alongside 16S rRNA gene amplicon sequencing. We found that gut metabolomes closely mirrored microbial composition, especially for the LC–MS/MS metabolomics data (Fig. 1A). This indicates that these microbial communities differ on the chemical level, suggesting that differences in microbial taxonomy lead to differences in function. Further evidence for this conclusion is the observation that related host species with distinct microbiomes also had distinct metabolomes, such as in the case of the zebras and rhinoceroses that were housed and fed together in the same enclosure. This strongly suggests that the differences in the metabolomes are due to differences in microbial metabolism, as opposed to gut morphology or diet. These results are supported by the recent finding that almost half of the metabolites detected in mouse stool did not appear in samples from germ-free mice, and were therefore likely microbial in origin [65]. However, an important caveat to this study is that it remains unclear how differences in mammalian host physiology and metabolism contribute to this variance: although zebras and rhinoceroses are both members of the order Perissodactyla, they are estimated to have diverged approximately 54 million years ago [90], ample time for differences to arise which could lead to differing intestinal metabolites. An additional caveat is that all samples in this study were collected from captive animals, from one zoological center. Previous studies on paired samples from wild and captive animals [91, 92] have shown that captivity has varying effects on the microbiome in different species, while possible effects on the metabolome remain unknown.

The mammalian gut metabolomes exhibited a strong degree of phylosymbiosis, with the relationships between the metabolomes closely recapitulating the phylogenetic tree of their mammalian hosts (Fig. 1B). It has been previously shown that phylosymbiosis can affect the function and phenotype of the ecosystem:, transplanting gut microbiomes between closely related mouse species caused a loss of fitness, measured by a decrease in digestibility of dry food [12]. This loss of fitness indicates that even these similar microbial communities from very closely related species were not in fact functionally redundant. In the current study, the finding that the gut metabolome closely mirrored the mammalian host phylogeny is indicative of such functional phylosymbiosis, and to the best of our knowledge, this is the first time that this phenomenon has been shown through analysis of metabolomics data. However, some of this signal may be due to host metabolites, which also could have a strong host phylogenetic signal. The complex interplay between host and microbial metabolites in the gut ecosystem makes it very difficult to differentiate between the two in fecal metabolomics studies, and determining the likely source of metabolites remains an open challenge in the field.

When we examined the metabolic dissimilarity across samples based on predicted functional annotations, we found that the degree of dissimilarity between samples in the LC–MS/MS metabolomics data was much greater than predicted by functional annotation, and was comparable to the dissimilarity of the microbial composition (Fig. 1C). In contrast, the dissimilarity between samples in the GC–MS metabolomics data was lower, closer to the levels based on predicted functional annotations (Fig. 1C). The LC–MS/MS metabolomics platform detects semi-polar metabolites and lipids up to 1500 Da and is therefore likely to detect rare, specialized metabolites that are more likely to be specific to certain microbial groups. On the other hand, the GC–MS metabolomics platform detects small, polar metabolites, including many of the fundamental building blocks of primary metabolism, for example, amino acids, SCFA, and sugars, that are common across most organisms. Therefore, it is not surprising that the GC–MS data showed a relatively low dissimilarity across samples, possibly reflecting a convergence of core metabolic functions. The predicted functional annotations are also likely to cover more abundant, shared functions, such as those in primary metabolism [16], and they showed a similar low dissimilarity across samples, as has been previously shown [21]. What the functional annotation may miss, however, are more rare functions involving specialized metabolites, which were indeed diverse and different across microbial species, as we found in the analysis of the LC–MS/MS metabolomics data. This is especially true for non-human mammalian microbiomes, which are less thoroughly studied and harder to functionally annotate than human microbiome samples, especially for predicted profiles extrapolated from 16S rRNA gene amplicon sequencing [32].

A key advantage of examining metabolic function through an untargeted metabolomics approach is that it is possible to quantify overall chemical diversity, thus avoiding biases stemming from relying on what is annotated. Additionally, this work highlights the importance of casting as wide a net as possible in metabolomics analysis, as the differences between the GC–MS and LC–MS/MS metabolomics datasets illustrate. However, an important caveat is that the degree of dissimilarity between samples for taxonomy has a clear dependence on the scale examined (e.g., samples are more dissimilar on the strain level than if categorized at the genus or family level) [21]. The metabolomics data here is measured at the single metabolite level and is therefore at a very fine resolution, but the same would presumably hold true at the chemical level if untargeted, unannotated metabolomics data could be accurately grouped into meaningful chemical families. Progress has been recently made in developing new chemical grouping algorithms that may make such analyses possible in the future [93, 94].

Next, we examined the metabolites driving the differences between animal gut metabolomes. For the LC–MS/MS metabolomics data, out of a total of over 200 metabolites enriched in different animal groups, we were able to putatively identify 74 metabolites using molecular networking (Fig. 2A, B). Some of these molecules belong to the interface between the host and the microbial community, including microbial modifications of host compounds such as bile acids and of dietary compounds such as triterpenoids. In the GC–MS metabolomics data, 14 out of 18 differential metabolites could be annotated. Since primary metabolism pathways are better annotated than those involved in specialized metabolism, most of these metabolites could be putatively linked to specific microbial pathways. The differential metabolites included the products of different microbial degradation pathways, such as fermentation products like SCFA, lactate, and succinate (Fig. 3A, B). We also observed degradation products of substrates expected to be enriched in different dietary groups, including biogenic amines resulting from amino acid decarboxylation in Carnivora (Fig. 3C), and an aromatic product of plant polyphenol degradation in Perissodactyla and Proboscidea (Fig. 3D). These results are in agreement with a previous mammalian metagenomics study which observed the enrichment of amino acid degradation enzymes in carnivores, and specifically predicted elevated succinate production, a prediction verified by the data shown here [14]. These findings support the hypothesis that these microbial communities are indeed adapted to their mammalian host environments on the functional level. However, an important limitation of this study is that the majority of peak features found here could not be annotated or linked to any specific host or microbial pathway, and even those that could be putatively identified were not conclusively verified, for example by comparing to a commercial standard. Future studies are needed to validate and expand upon these results, for example using targeted metabolomics analysis for specific differential metabolites together with metagenomics and/or transcriptomics data, as well as in vitro experiments to validate specific chemical transformations in fecal communities.

A possible explanation for the enrichment of certain metabolites is that over the course of evolutionary history, microbes have diversified due to pressures stemming from both the host and the microbial community, sometimes resulting in beneficial interactions between the two [11]. Microbial utilization of certain classes of indigestible host dietary compounds has been documented for example in the enrichment of specialized seaweed-degrading enzymes in the microbiota of Japanese individuals [95, 96], and in microbial catabolism of plant-derived toxins [97] such as the reduction of the drug digoxin by E. lenta [98, 99]. Here, we observe probable products of microbial catabolism of plant flavonoids, resulting in the enrichment of a phenolic breakdown product, as well as modifications of plant triterpenoid derivatives. Some microbial metabolites directly affect the host, as has been extensively documented for microbial fermentation products such as the SCFA highlighted here, especially propionate and butyrate, which are absorbed by the host and are known to affect human health [71, 100]. The microbial modifications of bile acids discussed here are also critical to host health, affecting host bile acid regulation as well as the incidence of liver cancer and the triggering of microbial pathogenicity [101, 102]. New host-microbe metabolic interactions are being discovered for lipids as well: two recent studies reported that microbes perform extensive conversions of dietary sphinganine to sphingolipids in the gut [103] and that such microbial sphingolipids are then incorporated in host signaling pathways and affect ceramide metabolism [104]. Overall, these findings lay the groundwork for further research into the complex interplay of host and microbe metabolic exchange.

In summary, in this study, we  found that mammalian metabolomes are chemically diverse and strongly linked to microbiome composition, and that metabolome composition is further correlated with the phylogeny of the mammalian host. We show that mammalian gut microbiomes do not exhibit high levels of functional redundancy on the level of the metabolome. Specific metabolites enriched in different host species were found to be associated with the host-microbe interface, including the modification and degradation of host and dietary compounds. These findings represent a first step towards unraveling the chemical ecology of the mammalian microbiome, and towards a better understanding of the origin and relevance of the vast gut microbial diversity found across mammals.

## Supplementary information

Supplemental material

Table S1

Table S2

Table S5

## Data Availability

An R script including the code to generate figures and statistical analysis for this work is available on GitHub: https://github.com/RachelGregor/ZooMicrobiomeMetabolome. Sequencing data are provided at the NCBI (SRA) database under the study accession code PRJNA693262 (https://www.ncbi.nlm.nih.gov/bioproject/PRJNA693262/). All of the mass spectrometry data used in preparation of this manuscript are publicly available at the MassIVE repository at the UCSD Center for Computational Mass Spectrometry website (massive.ucsd.edu) and linked to the Reanalysis of Data User (ReDU) interface (https://redu.ucsd.edu/) [62], and molecular networking analysis results are available on the GNPS website (gnps.ucsd.edu). For the LC–MS/MS data, the dataset accession number is MSV000086131 (10.25345/C55R2D). The LC–MS/MS networking analysis is available at https://gnps.ucsd.edu/ProteoSAFe/status.jsp?task=22633c67c0cb4da2a3b0b0fd1f1576f1. The NAP analysis is available at https://gnps.ucsd.edu/ProteoSAFe/status.jsp?task=c53c21f592014e7cadb8efaaf6046776. The MolNetEnhancer analysis is available at https://gnps.ucsd.edu/ProteoSAFe/status.jsp?task=563807dbb20e442c8ea561d4e98d44a4. For the GC–MS data, the dataset accession number is MSV000083859 (10.25345/C5W63S). The GC–MS spectral deconvolution results are available at https://gnps.ucsd.edu/ProteoSAFe/status.jsp?task=150f45ec05924c61af8d9ff99c8f2a87. The GC–MS networking analysis is available at https://gnps.ucsd.edu/ProteoSAFe/status.jsp?task=babcee3477644edc92a6fc2a191aded8.
