# Supplementary material for: Mammalian gut metabolomes mirror microbiome composition and host phylogeny
Source: ISME J. 2021 Dec 13;16(5):1262–74. doi: 10.1038/s41396-021-01152-0 (PMC9038745; doi:10.1038/s41396-021-01152-0)
Supplement: Supplementary file 1 — Supplemental material [file 41396_2021_1152_MOESM1_ESM.pdf]

Supplementary information for

Mammalian gut metabolomes mirror microbiome composition and host phylogeny

Rachel Gregor[1,2], Maraike Probst[2,3], Stav Eyal[2,3], Alexander Aksenov[4], Goor Sasson[2,3], Igal Horovitz[5], Pieter C. Dorrestein[4,6,7,8], Michael M. Meijler[1,2]*, Itzhak Mizrahi[2,3]*

[1]Department of Chemistry, Ben-Gurion University of the Negev, Be'er Sheva, Israel

[2]National Institute of Biotechnology in the Negev, Ben-Gurion University of the Negev, Be'er Sheva, Israel

[3]Department of Life Sciences, Ben-Gurion University of the Negev, Be'er Sheva, Israel

[4]Collaborative Mass Spectrometry Innovation Center, Skaggs School of Pharmacy and Pharmaceutical Sciences, University of California San Diego, La Jolla, CA, USA

[5]The Zoological Center Tel Aviv-Ramat Gan, Ramat Gan, Israel

[6]Center for Microbiome Innovation, University of California San Diego, La Jolla, CA, USA

[7]Department of Pharmacology, School of Medicine, University of California San Diego, La Jolla, CA, USA

[8]Department of Pediatrics, University of California San Diego, La Jolla, CA, USA

*Corresponding authors: Michael M. Meijler (meijler@bgu.ac.il) and Itzhak Mizrahi (imizrahi@bgu.ac.il)

This PDF file includes:

Tables S1 to S8 (Tables S1, S2, and S5 are additional Excel files)

Figures S1 to S17

Supplementary methods

Supplementary tables

Table S1. Excel file containing metadata of all samples.

Table S2. Excel file containing all significantly enriched metabolites and annotations. The file includes four tabs: annotated LC-MS/MS (74 peak features which could be classified/annotated); unidentified LC-MS/MS features (156 additional unidentified significant peak features); GC-MS (18 significant peak features); and features removed from GC-MS (7 peak features removed from analysis).

Table S3. Mantel correlations between metabolomics and microbial composition datasets for: median datasets in which n=1 for each species; random subsets in which samples were randomly subsampled per host species, for a total of maximum of n=1-5 samples per species (for species with fewer than n individuals sampled, all samples were included); intraspecies comparisons for species with at least 10 individuals sampled (Bray–Curtis dissimilarity, Pearson correlation).

	16S rRNA gene sequencing vs. LC-MS/MS			16S rRNA gene sequencing vs. GC-MS			LC-MS/MS vs. GC-MS		
	r statistic	*p* value		*r* statistic	*p* value		*r* statistic	*p* value	
Median (n=1)	0.62	0.0001	***	0.13	0.06		0.58	0.0001	***
Random (n=1)	0.57	0.0001	***	0.03	0.39		0.42	0.0001	***
Random (n≤2)	0.65	0.0001	***	0.15	0.001	**	0.50	0.0001	***
Random (n≤3)	0.62	0.0001	***	0.17	0.0001	***	0.55	0.0001	***
Random (n≤4)	0.60	0.0001	***	0.19	0.0001	***	0.54	0.0001	***
Random (n≤5)	0.59	0.0001	***	0.18	0.0001	***	0.52	0.0001	***
Zebras (n=16)	0.30	0.052		-0.03	0.47		0.20	0.12	
Chimpanzees (n=10)	0.27	0.072		0.61	0.045	*	-0.11	0.72	
Rhinoceroses (n=13)	0.76	0.002	**	0.39	0.085		0.74	0.008	**

Table S4: PERMANOVA (Adonis) results based on Bray–Curtis dissimilarity, including sex. Samples n=82 (19 samples for which sex was not recorded, including all 16 zebra samples, were removed from the analysis). Permutations n=999. Abbreviations: Df.= degrees of freedom; Sqs= squares.

16S rRNA gene amplicon sequencing

	Df.	Sums of Sqs.	Mean Sqs.	PseudoF	R^2	*p* value	
Diet	2	5.12	2.56	10.90	0.14	0.001	***
Gut morphology	2	3.47	1.73	7.37	0.09	0.001	***
Mammalian order	2	5.71	2.86	12.16	0.15	0.001	***
Host species	16	8.90	0.56	2.37	0.24	0.001	***
Sex	1	0.25	0.25	1.08	0.01	0.31	
Collection time	1	0.24	0.24	1.01	0.01	0.42	
Residuals	57	13.39	0.23		0.36		
Total	81	37.08			1		

LC-MS/MS

	Df.	Sums of Sqs.	Mean Sqs.	PseudoF	R^2	*p* value	
Diet	2	5.24	2.62	19.35	0.20	0.001	***
Gut morphology	2	2.96	1.48	10.93	0.11	0.001	***
Mammalian order	2	3.91	1.96	14.45	0.15	0.001	***
Host species	16	6.19	0.39	22.85	0.23	0.001	***
Sex	1	0.12	0.12	0.91	0.00	0.53	
Collection time	1	0.29	0.29	2.15	0.01	0.009	**
Residuals	57	7.72	0.14		0.29		
Total	81	26.44			1		

GC-MS

	Df.	Sums of Sqs.	Mean Sqs.	PseudoF	R^2	*p* value	
Diet	2	0.88	0.44	12.35	0.16	0.001	***
Gut morphology	2	0.31	0.15	4.32	0.05	0.001	***
Mammalian order	2	0.88	0.44	12.33	0.16	0.001	***
Host species	16	1.46	0.09	2.57	0.26	0.001	***
Sex	1	0.05	0.05	1.30	0.01	0.23	
Collection time	1	0.04	0.04	1.04	0.01	0.38	
Residuals	57	2.03	0.04		0.36		
Total	81	5.64			1		

Table S5. Excel file with the PERMANOVA (Adonis) results based on Bray–Curtis dissimilarity for subsets of data. Samples were randomly subsampled per host species, for a total of maximum of n=1–5 samples per species (for species with fewer than n individuals sampled, all samples were included). Permutations n=999. Abbreviations: Df.= degrees of freedom; Sqs= squares.

Table S6. Mantel correlations between host patristic distances and metabolomics and microbial composition datasets (Bray Curtis dissimilarity, Spearman correlation).

Host patristic distances vs.:	*r* statistic	*p* value	
16S rRNA gene amplicon sequencing	0.45	0.0001	***
LC-MS/MS	0.28	0.004	**
GC-MS	0.09	0.23	

Table S7. Mass differences and annotations in the triterpenoid molecular network.

Δ m/z (Da)	Annotation
0.000	Structural isomer or chromatography artifact
± 2.016	± H_2 (hydrogenation/dehydrogenation)
± 13.979	O ↔ H_2 (oxidation and H_2 elimination, or vice versa)
± 15.995	± O (oxygen gain/loss)
± 18.011	± H_2O (addition of H_2O across double bond or vice versa)
± 31.990	± 2O (2 oxygen gain/loss)
- 79.956	- SO_3 (desulfation only; two sulfated analogues were not significantly differential metabolites)

Table S8. Average acetate:propionate:butyrate ratios (±standard deviation).

	Acetate	Propionate	Butyrate
Carnivora (carn.)	49.5 (±9.5)	24.8 (±10.4)	25.6 (±9.0)
Carnivora (omni.)	72.6 (±4.6)	6.6 (±3.2)	20.8 (±4.6)
Primates (herb.)	51.6 (±2.2)	27.8 (±1.7)	20.6 (±2.0)
Primates (omni.)	56.6 (±9.2)	25.9 (±4.2)	17.5 (±6.2)
Proboscidea	53.0 (±4.4)	30.5 (±3.2)	16.5 (±2.1)
Perissodactyla	60.2 (±4.3)	29.1 (±3.0)	10.7 (±2.8)
Artiodactyla	68.2 (±3.9)	23.2 (±2.8)	8.6 (±2.8)

Supplementary figures

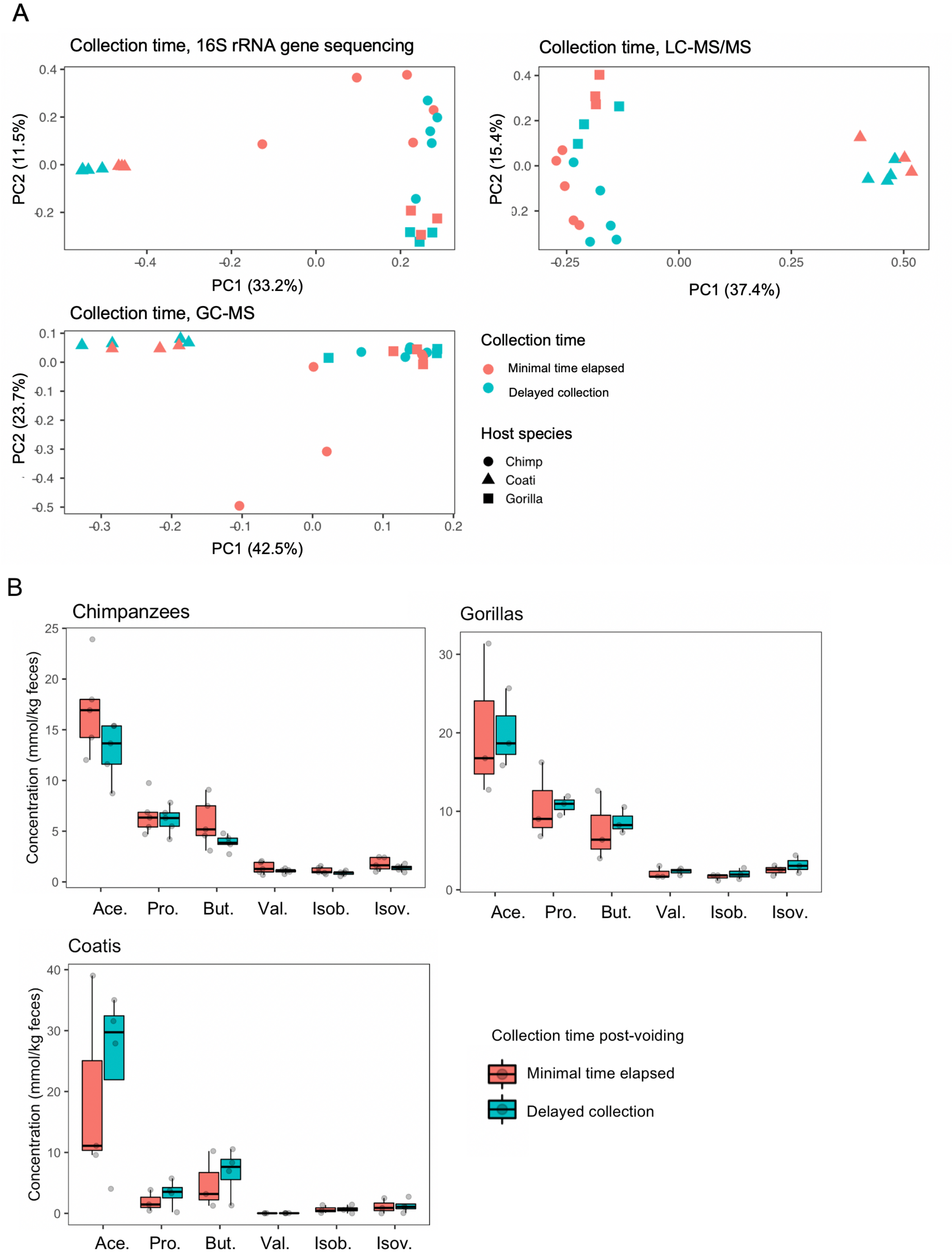

Fig. S1. Effect of collection times. Comparisons were made for three species for which the elapsed time between defecation and sample collection varied. Samples in the “delayed collection” category were

deposited at some point in the night and collected in the morning, before hardening, and the estimated time elapsed is 4-16 hours (additionally, one chimpanzee sample in this category was collected in the afternoon, and is estimated to fall into the same time range). Samples in the “Minimal time elapsed” category were either collected directly post-voiding (chimpanzees, gorillas), or 1-2 hours post-voiding (coatis). A. PCoA (Bray–Curtis dissimilarity) for each dataset. Primary clustering is by host species, not collection time. B. Comparison of SCFA levels in fecal samples with differing collection times. No overall trend of loss of signal was noted for these compounds. Full information on the collection times is available in Table S1.

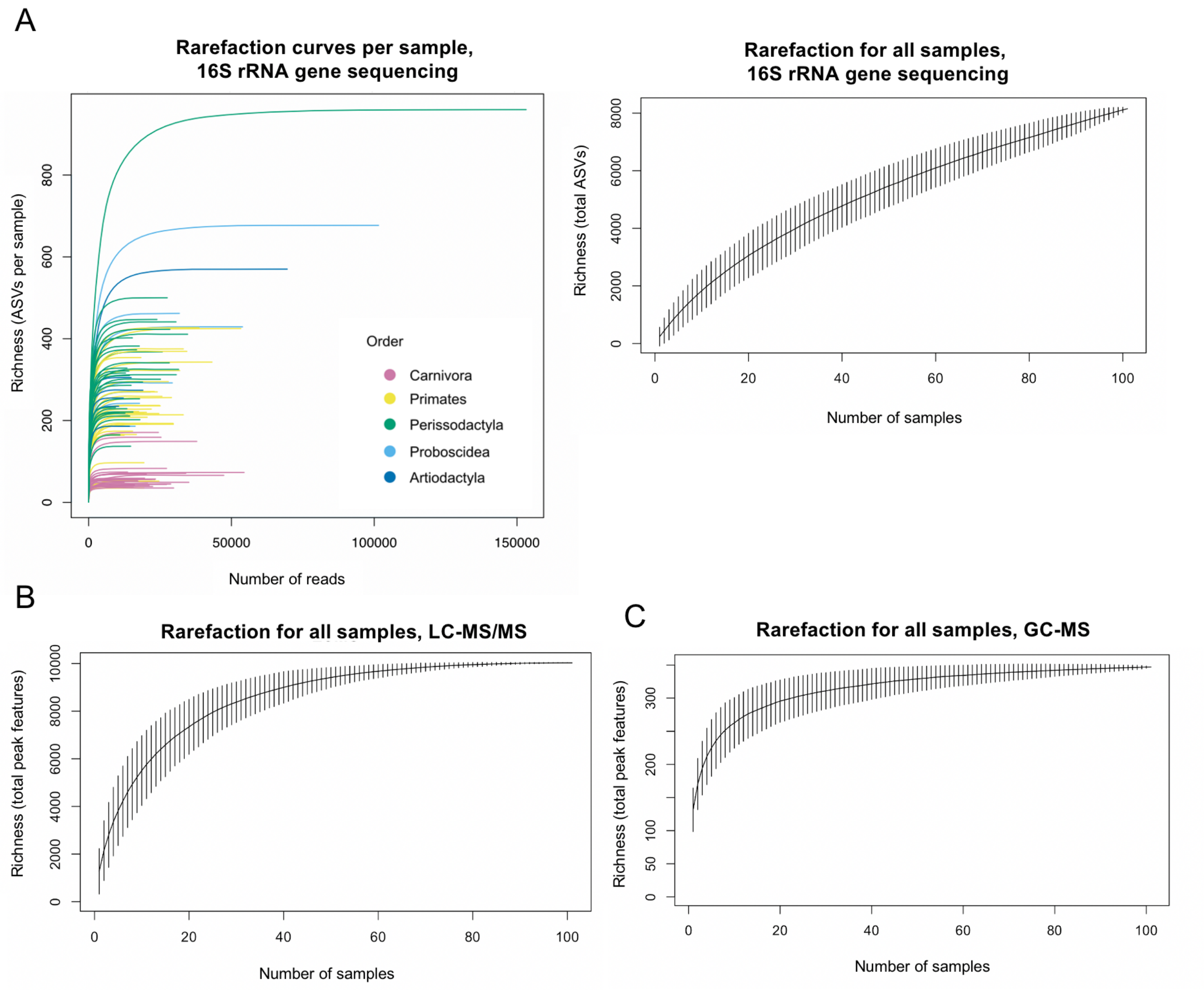

Fig. S2. Rarefaction curves for each dataset. A) 16S rRNA gene amplicon sequencing, left, distribution of reads and rarefaction curves per sample; right, rarefaction curve of all samples (500 permutations). B) LC-MS/MS and C) GC-MS rarefaction curves of all samples (500 permutations).

Richness per host species

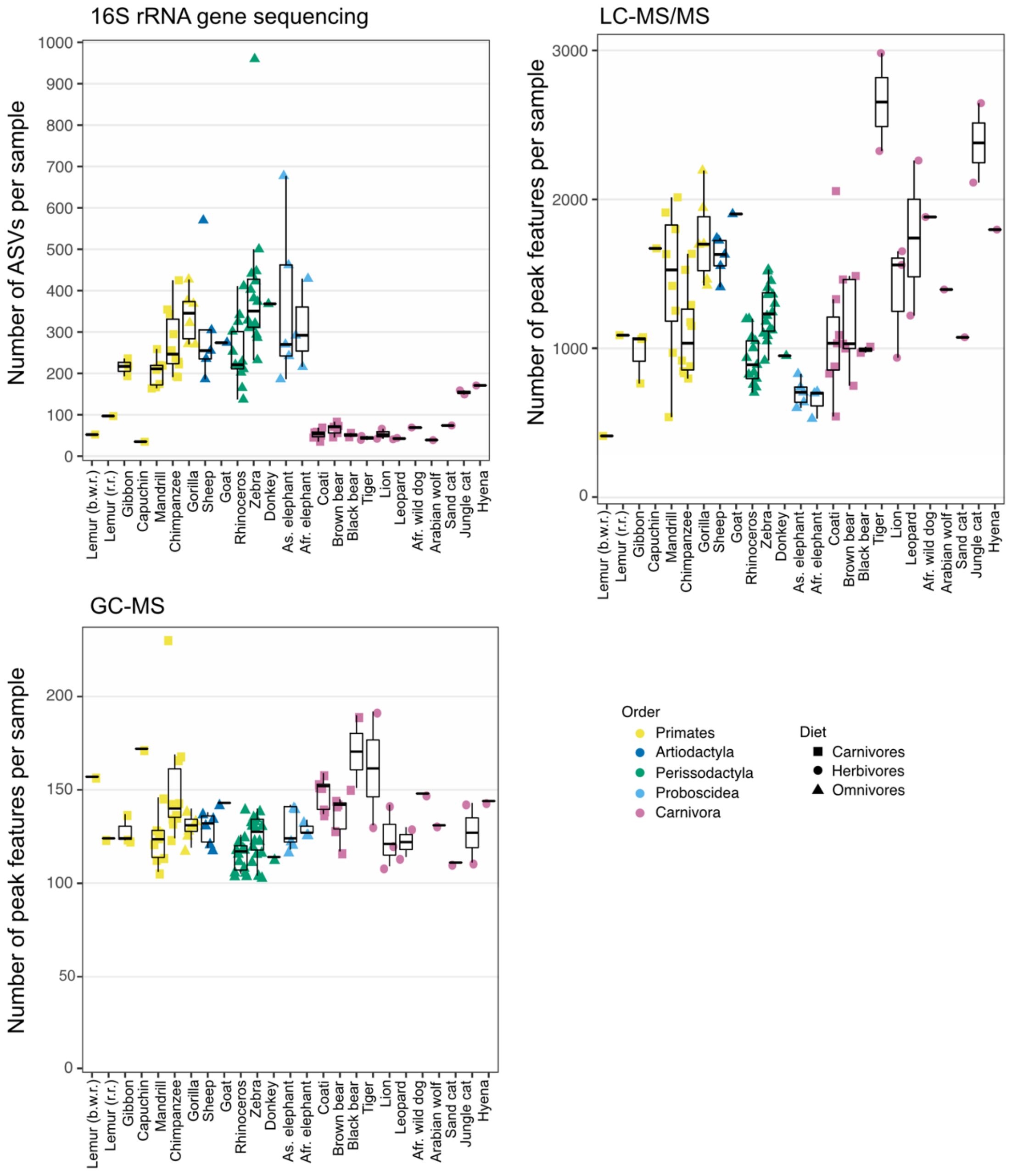

Fig. S3. Richness per host species of ASVs (16S rRNA gene amplicon sequencing) and peak features (LC-MS/MS and GC-MS).

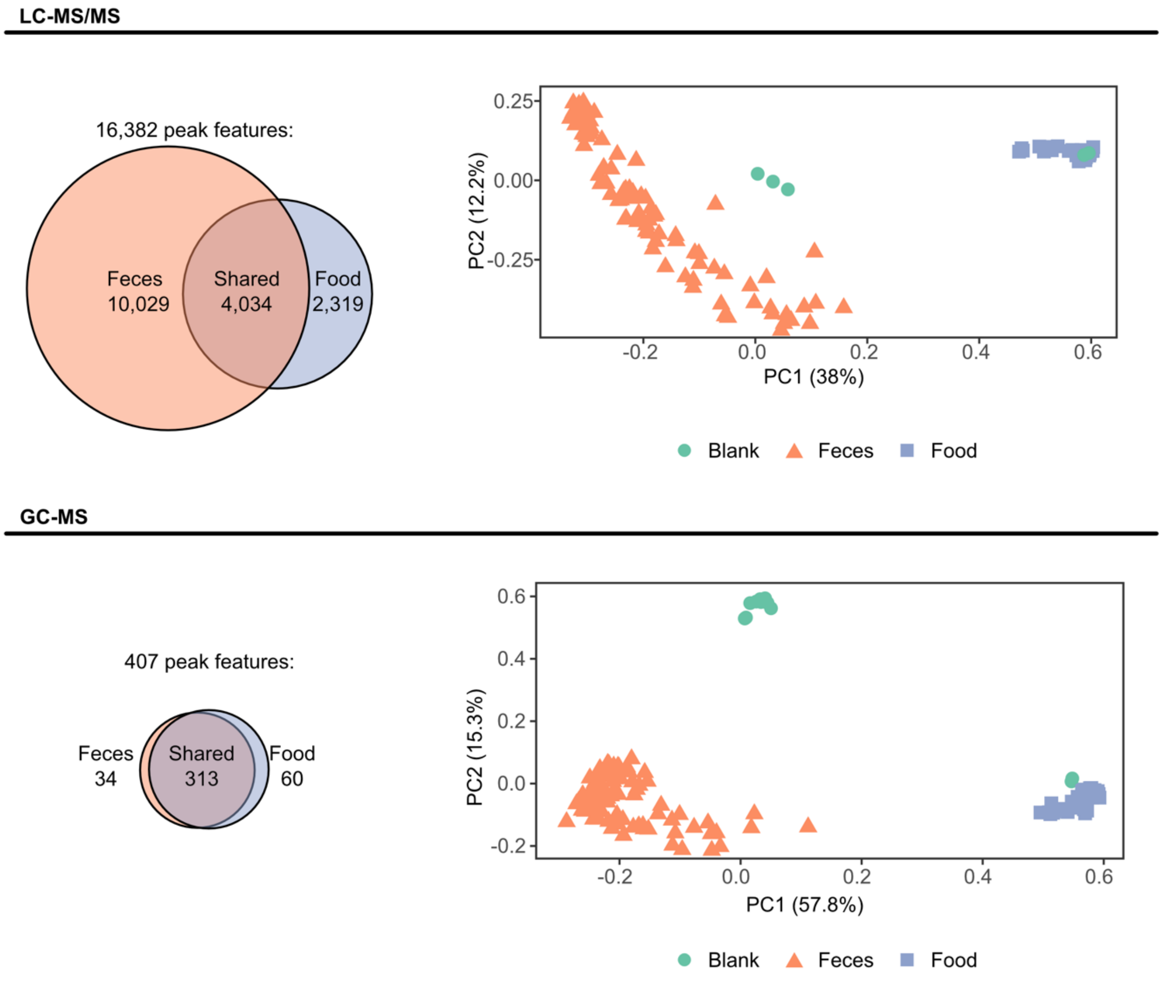

Fig. S4. Comparison of fecal and food metabolomes, for LC-MS/MS (top) and GC-MS (bottom) metabolomics data. Left, Venn diagrams describing the distribution of peak features across the fecal and food metabolomes. For the LC-MS/MS metabolomics data (top left), the shared peak features that were detected in both food and fecal samples were removed from the analysis, in order to minimize signal resulting solely from differences in undigested dietary compounds. The resulting peak list of 10,029 peak features was used for all further analyses. For the GC-MS metabolomics data (bottom left), over 75% of peak features were shared between the feces and food (see discussion in the Subjects and Methods section). Right, PCoA of samples based on the Bray–Curtis dissimilarity metric, showing a separation between food, fecal, and blank samples. Note: the two blank samples that cluster with the food samples represent background samples that underwent the *in vitro* digestion protocol.

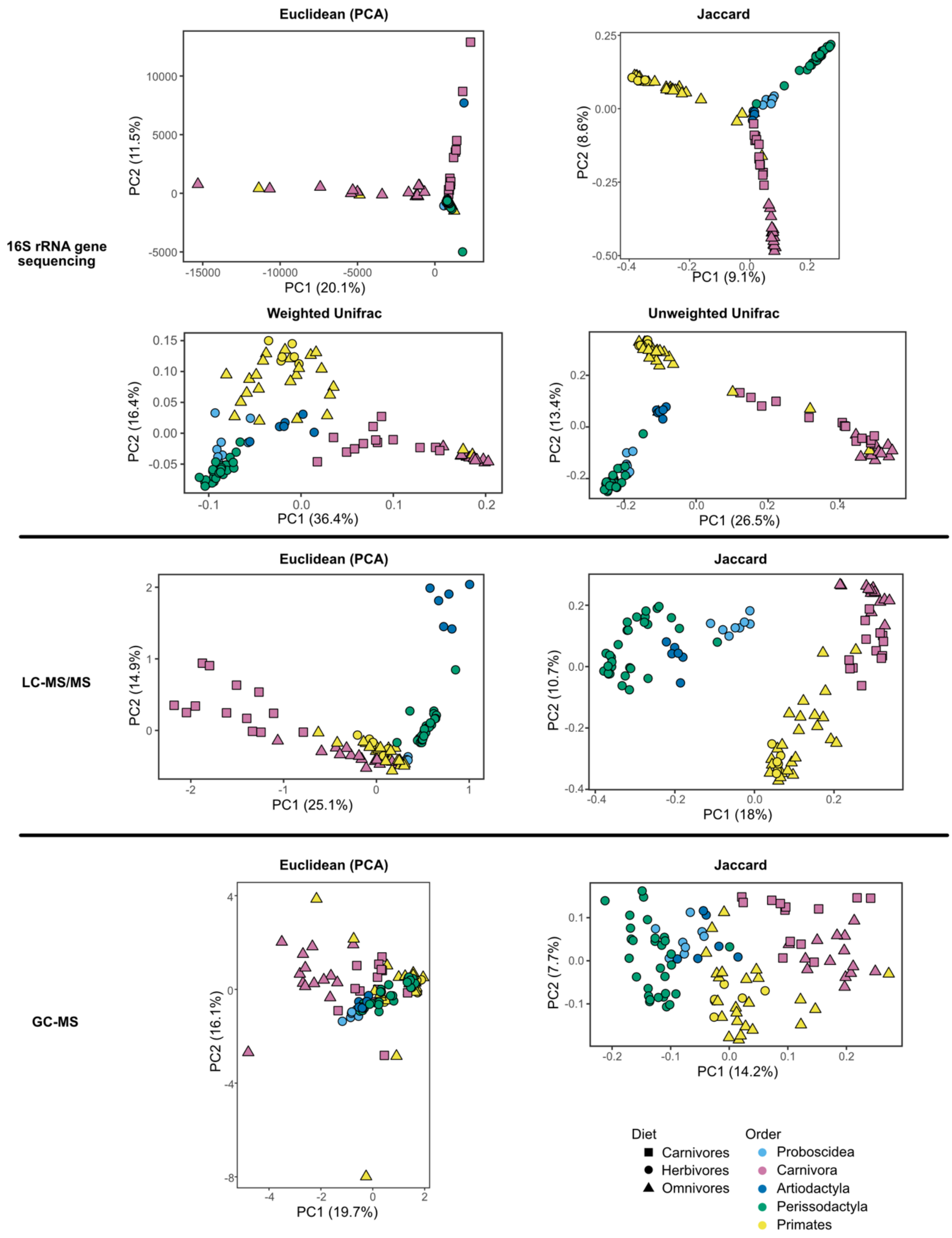

Fig. S5. Additional ordination metrics for the three data types: 16S rRNA gene amplicon sequencing, LC-MS/MS metabolomics and GC-MS metabolomics. For each data set, Euclidean distances (principal component analysis, PCA) and Jaccard dissimilarity (based on presence/absence) are shown, as well as for the microbial composition data, weighted and unweighted Unifrac dissimilarity, which take into account microbial phylogeny.

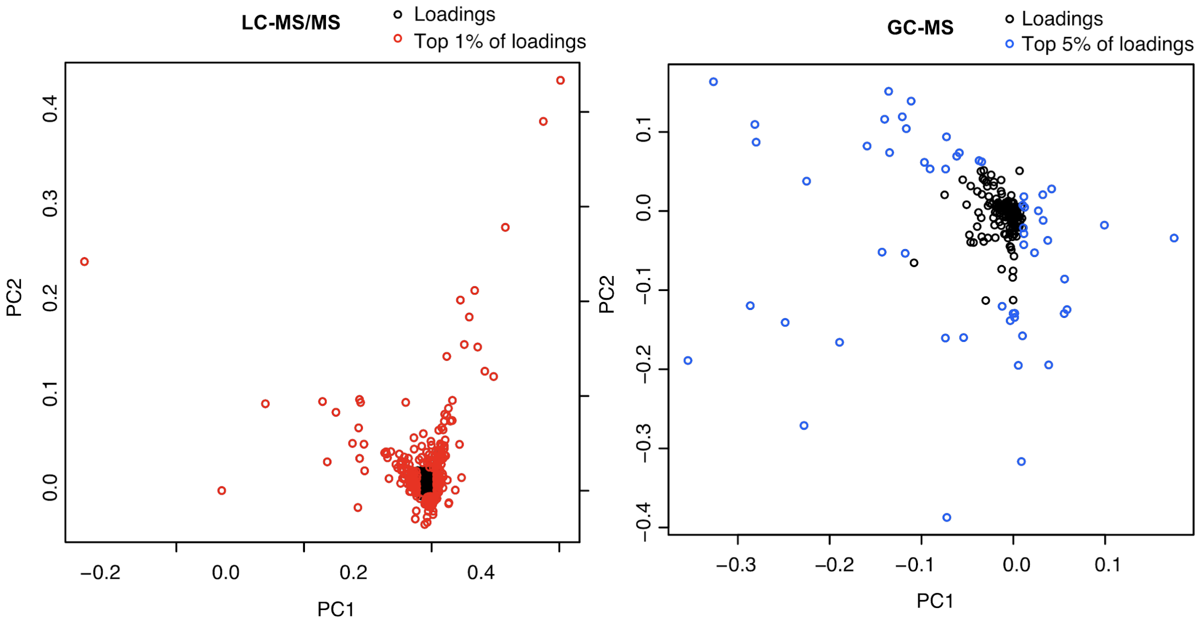

Fig. S6. The top and bottom percentiles of loadings of the PCA were extracted, in order to create a shortlist of candidate peak features for further statistical analysis. For the LC-MS/MS data (left), the top 5% of loadings included over 1,500 peak features, and so the list was further filtered to the top 1% of loadings (319 peak features). For the GC-MS data (right), the top 5% of loadings included 55 peak features.

A

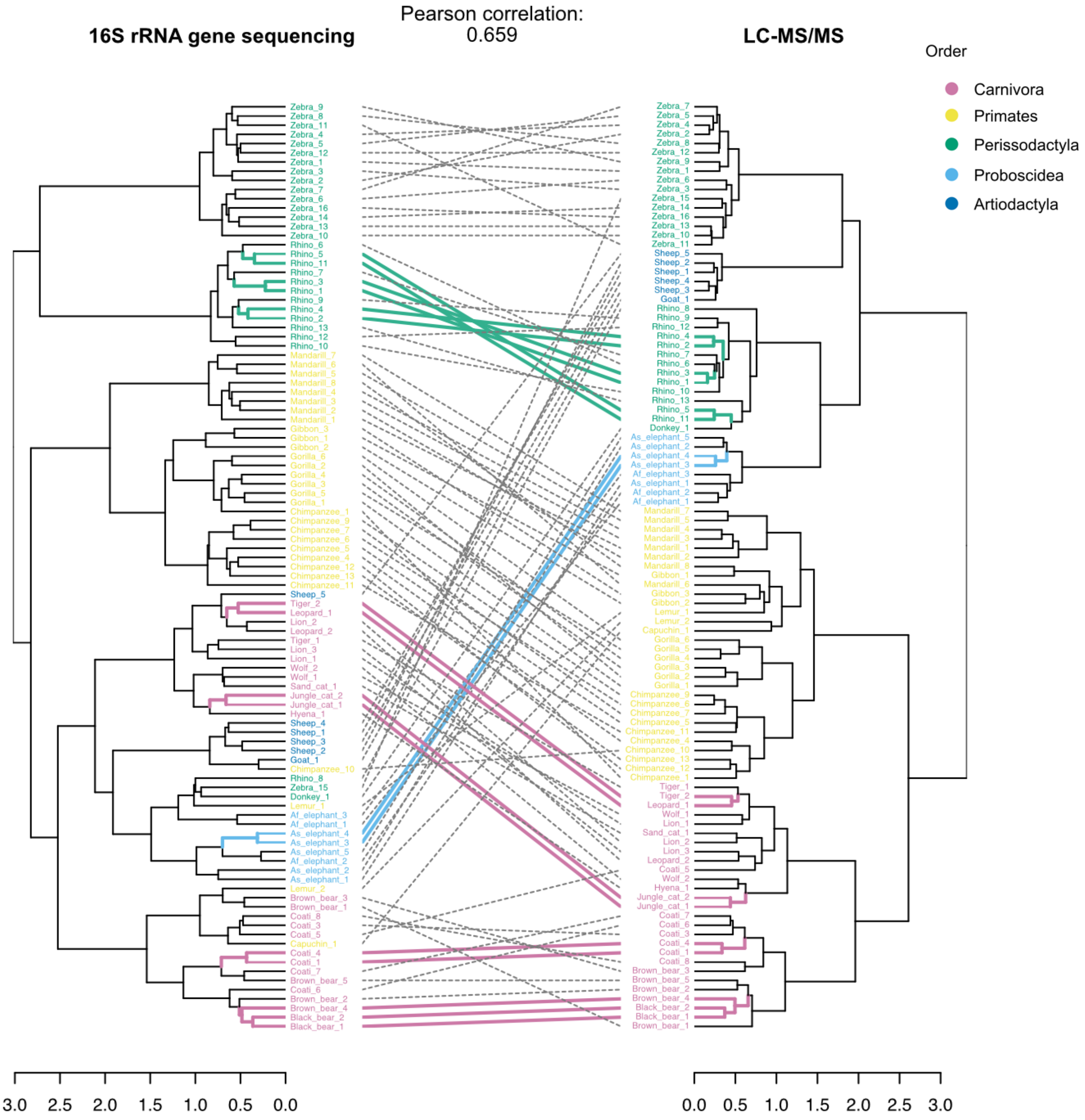
16S rRNA gene sequencing
Pearson correlation:
0.659
LC-MS/MS
Order
Carnivora
Primates
Perissodactyla
Proboscidea
Artiodactyla
3.0 2.5 2.0 1.5 1.0 0.5 0.0
0.0 0.5 1.0 1.5 2.0 2.5 3.0

B

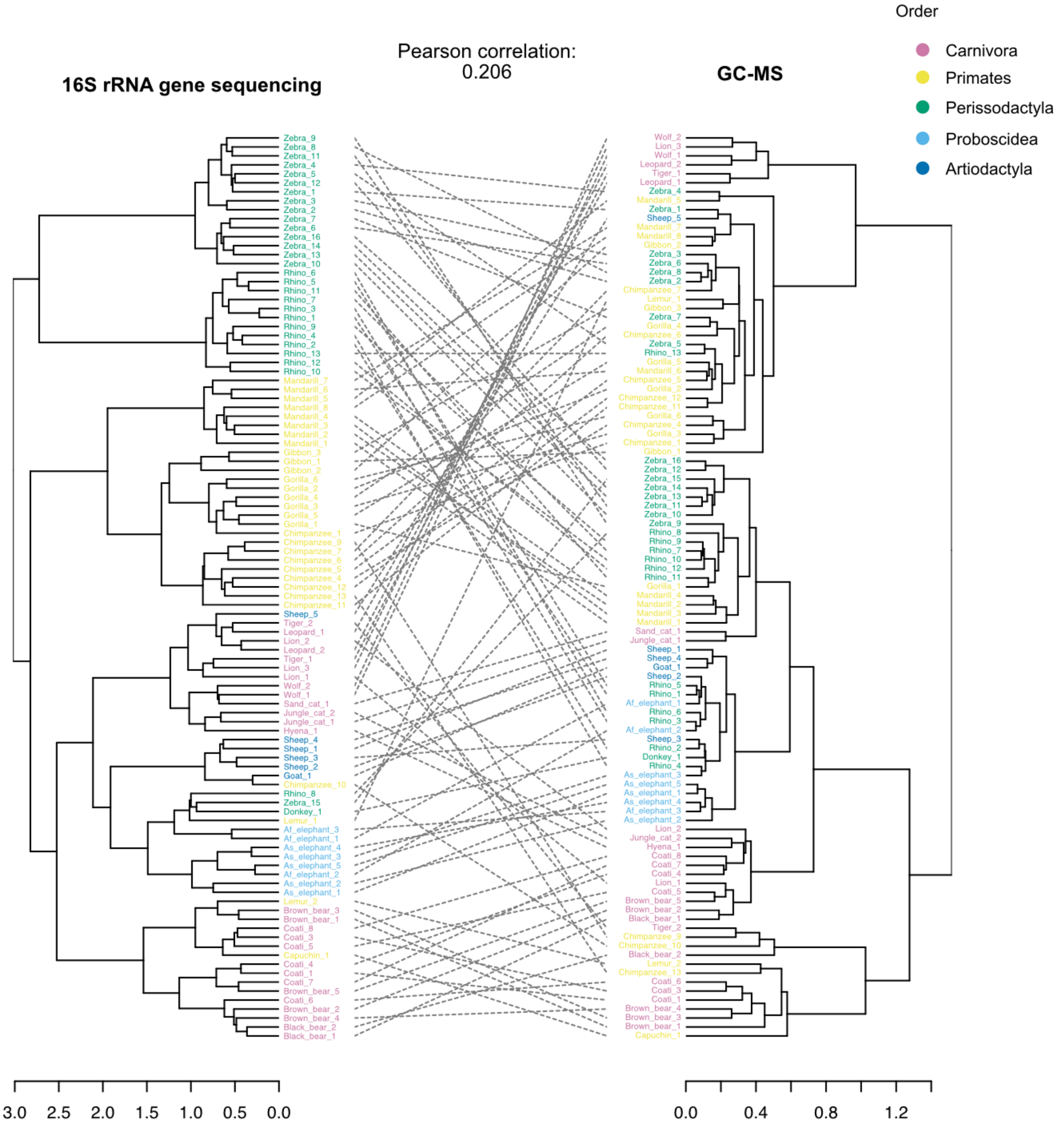
16S rRNA gene sequencing
Pearson correlation:
0.206
GC-MS
Order
Carnivora
Primates
Perissodactyla
Proboscidea
Artiodactyla
3.0 2.5 2.0 1.5 1.0 0.5 0.0
0.0 0.4 0.8 1.2

C

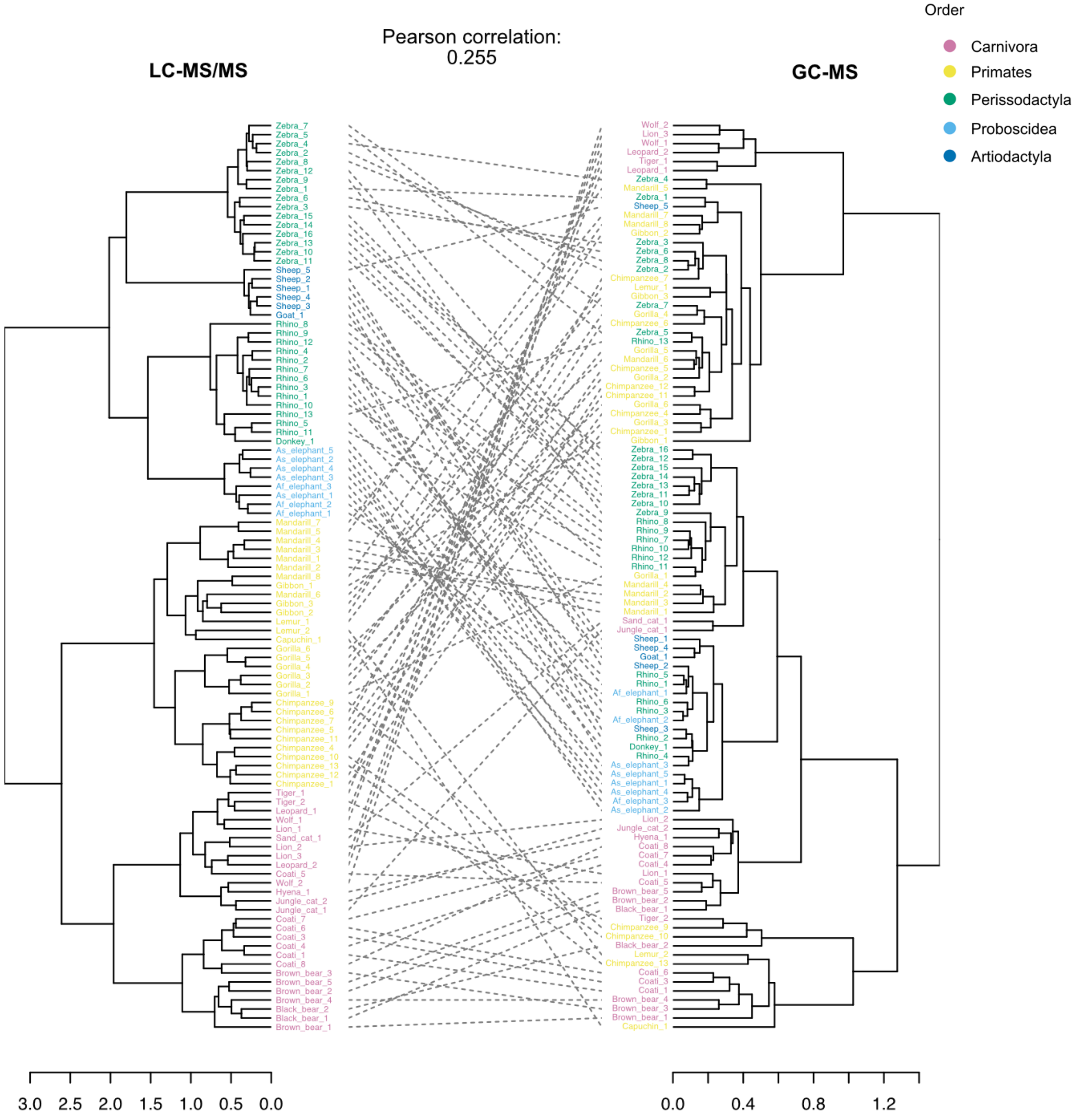

Fig. S7. Tanglegrams with all samples to compare between trees of the datasets (Bray–Curtis dissimilarity and Ward's hierarchical clustering method): A) 16S rRNA gene amplicon sequencing vs. LC-MS/MS; B) 16S rRNA gene amplicon sequencing vs. GC-MS; C) LC-MS/MS vs. GC-MS.

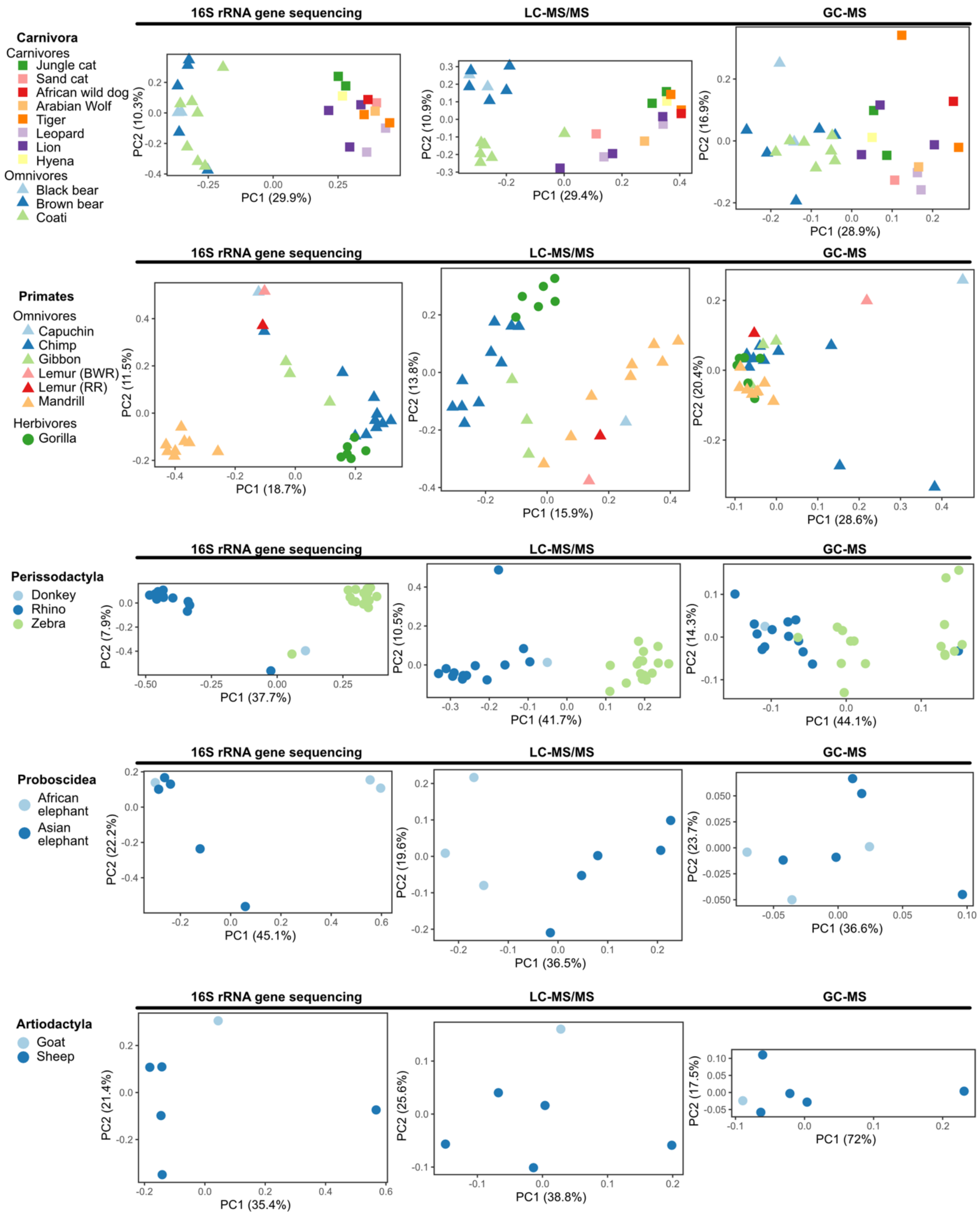

Fig. S8. PCoA of samples based on the Bray–Curtis dissimilarity metric for subsets by mammalian order, for the three data types: 16S rRNA gene amplicon sequencing, LC-MS/MS metabolomics data and GC-MS metabolomics data.

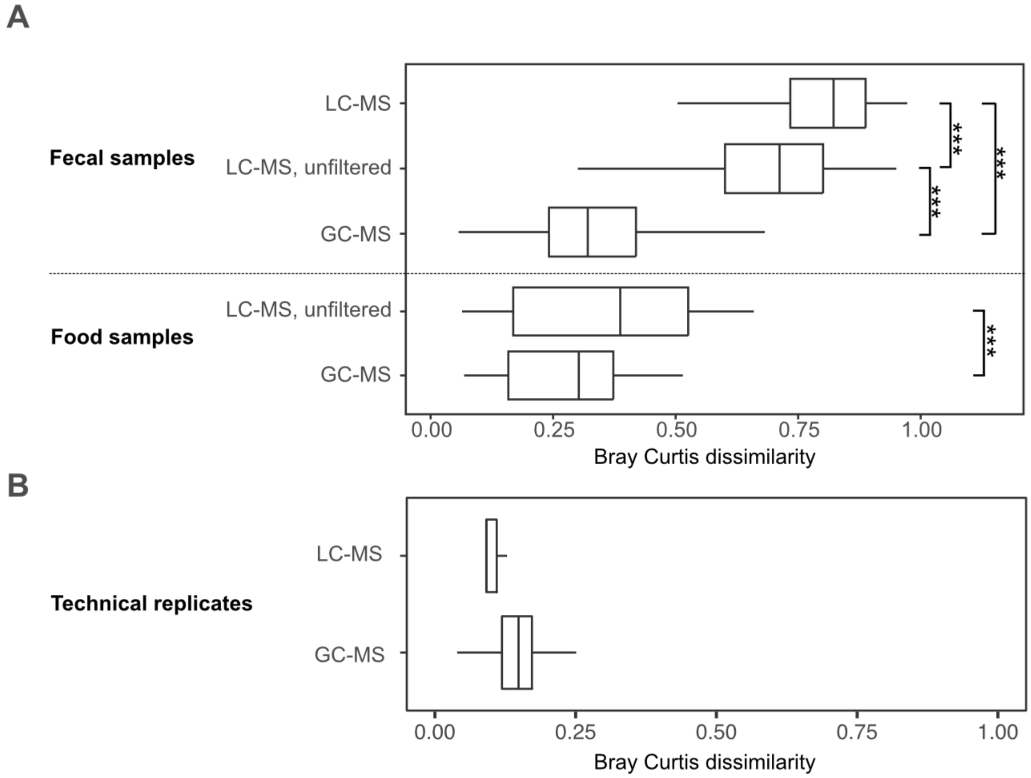

Fig. S9. A. Comparison of sample dissimilarity of fecal samples to food samples. For fecal samples, the LC-MS/MS metabolomes were highly dissimilar (median dissimilarity=0.823), while the GC-MS metabolomes were more similar to one another (median dissimilarity=0.321) (reproduced here from Fig. 1C). The GC-MS metabolomes included food metabolites, while the LC-MS/MS metabolomes were filtered; however, the unfiltered LC-MS/MS metabolome data was also highly dissimilar (median dissimilarity=0.713). For comparison, this difference was much smaller for the food samples (median dissimilarity for food LC-MS/MS metabolome=0.387; for food GC-MS metabolome=0.302), indicating that this difference is not inherent to the LC-MS/MS vs GC-MS analysis platforms, but rather is due to a biological difference in the metabolic composition of the fecal samples. B. Comparison of Bray Curtis dissimilarity for technical replicates run through the instrument run for LC-MS/MS (n=3, median dissimilarity=0.091) and for GC-MS (n=26, median dissimilarity=0.149) (more technical replicates were run for GC-MS due to a planned instrument service event during analysis which contributed to higher sample variance over time). For all panels, for the boxplots, the lower and upper hinges correspond to the first and third quartiles, and outliers are not shown. The significance was determined using one-way ANOVA analysis followed by post-hoc Tukey’s test, resulting in adjusted *p* values noted as follows: p<0.001= ***.

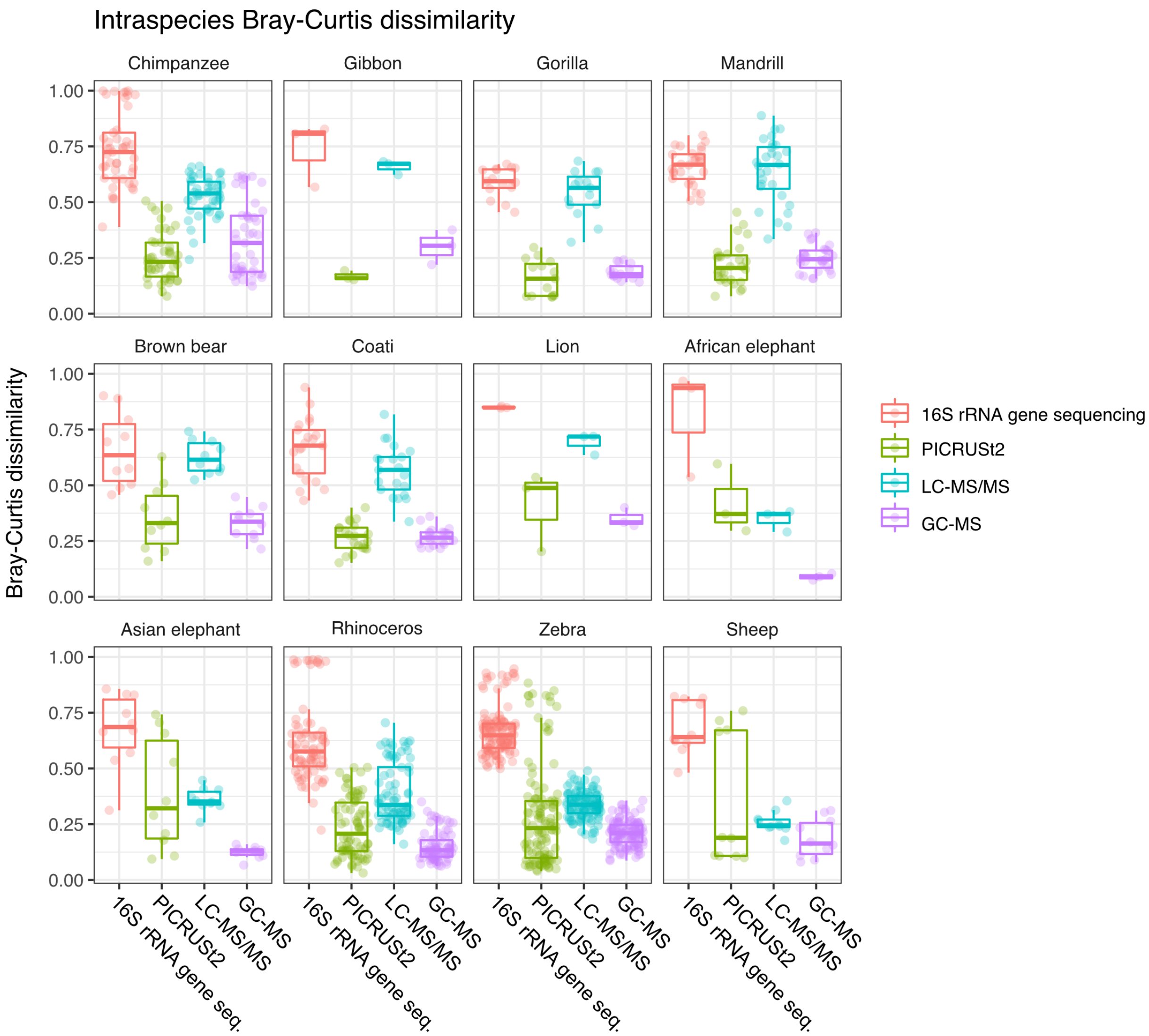

Fig. S10. Comparison of intraspecies Bray–Curtis dissimilarity for host species with more than 3 individuals sampled.

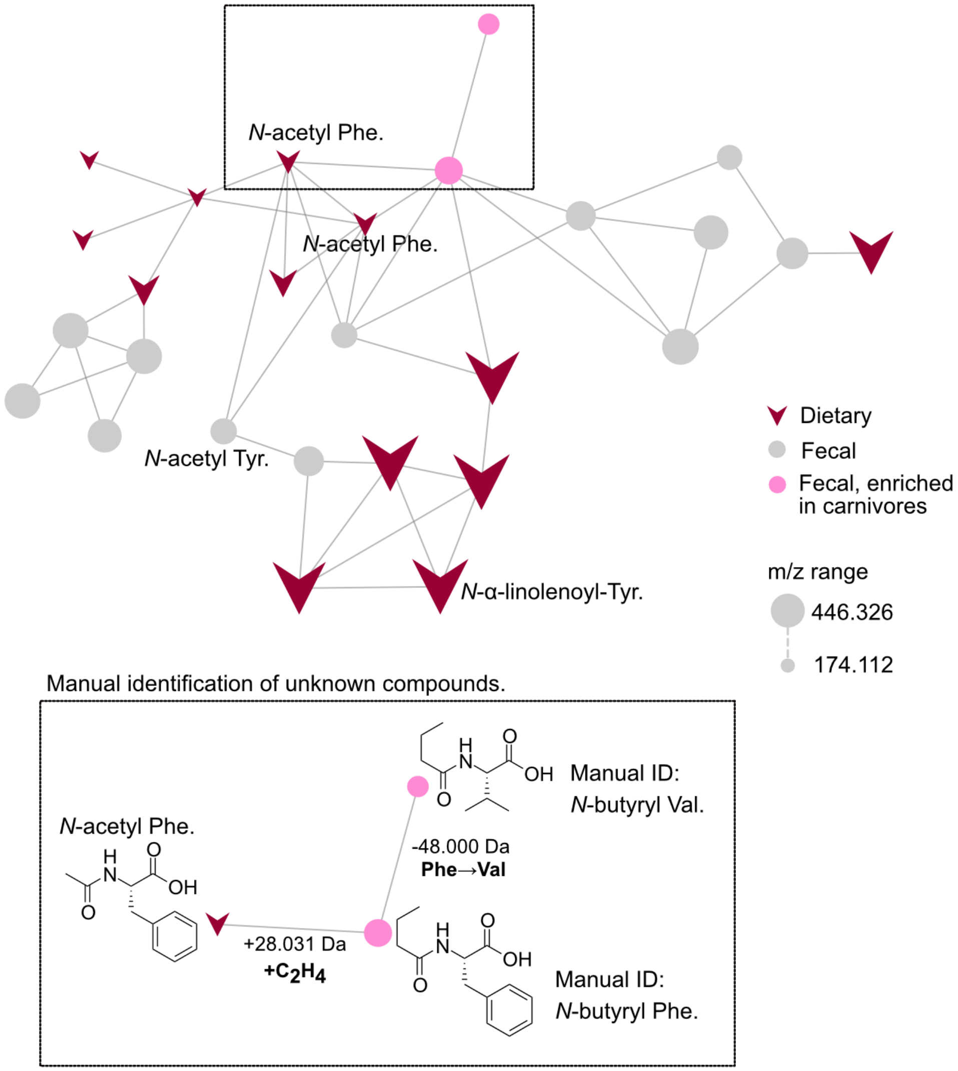

Fig. S11. Example of manually propagating library annotations using molecular networking. A subnetwork of *N*-acylated amino acids (top) contained two unidentified significantly enriched metabolites (pink). However, a neighboring node was identified as *N*-acetyl phenylalanine based on the GNPS spectral library search. As shown in the inset, it has a mass difference of +28.031 Da from one of the significantly enriched metabolites, which corresponds by exact mass to the extension of the chain length by two carbon units, likely *N*-butyryl phenylalanine. The mass difference to the second node corresponds to the substitution of valine for phenylalanine, likely *N*-butyryl valine. These putative identifications were then further verified by manually examining the fragmentation spectra and identifying characteristic valine and phenylalanine peaks. Note: we were not able to determine if the acylation corresponds to n-butyryl or a structural isomer such as isobutyryl.

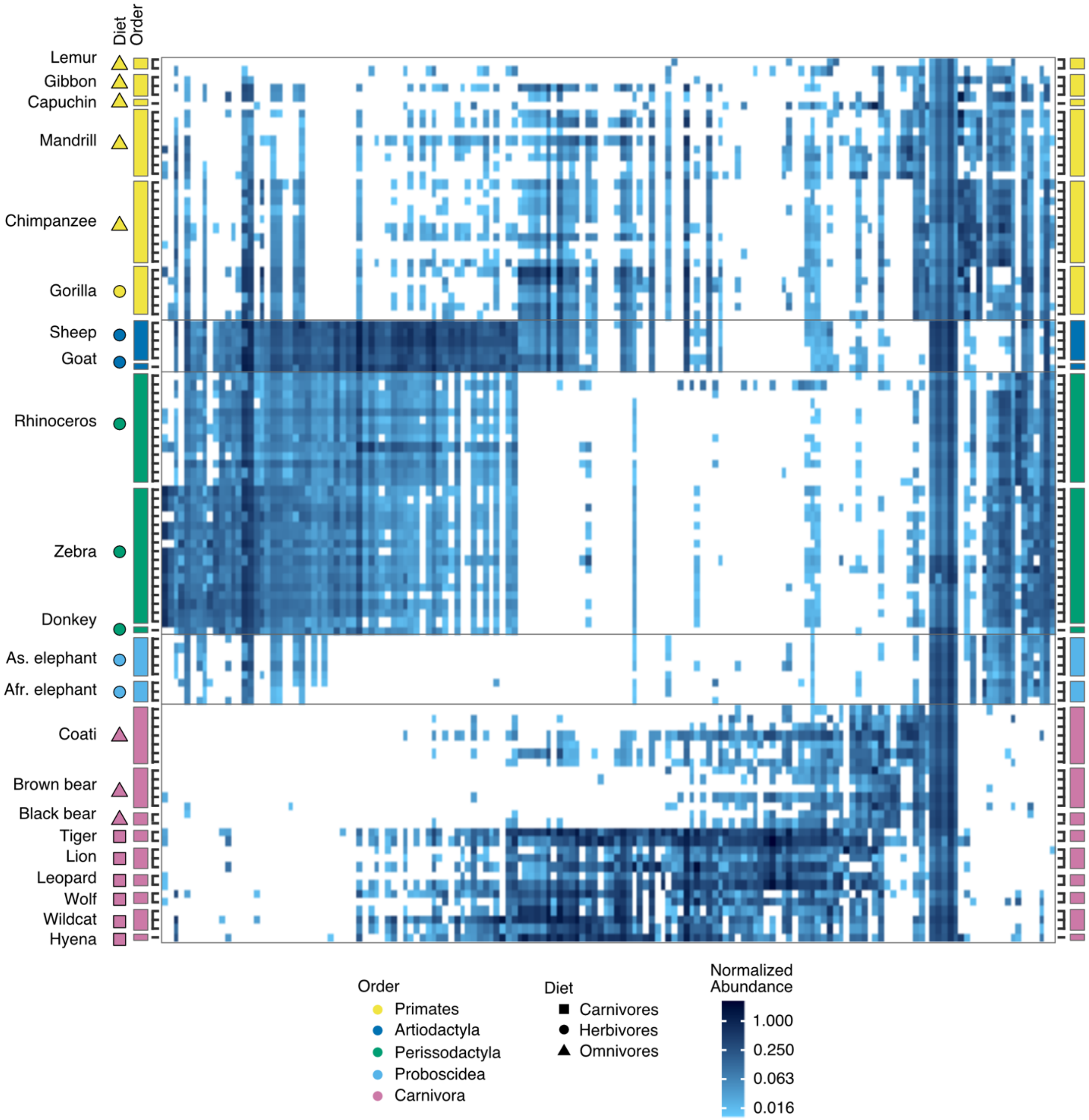

Fig. S12. Heatmap of 156 unidentified LC-MS/MS differential peak features. Peak features were clustered using non-metric multidimensional scaling and Bray–Curtis dissimilarity, then arranged by sample order as noted on left.

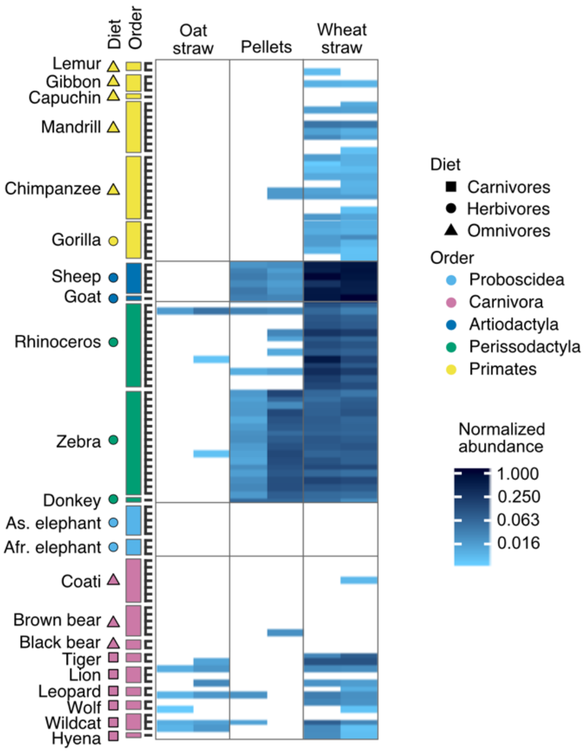

Fig. S13. Heatmap of dietary triterpenoids.

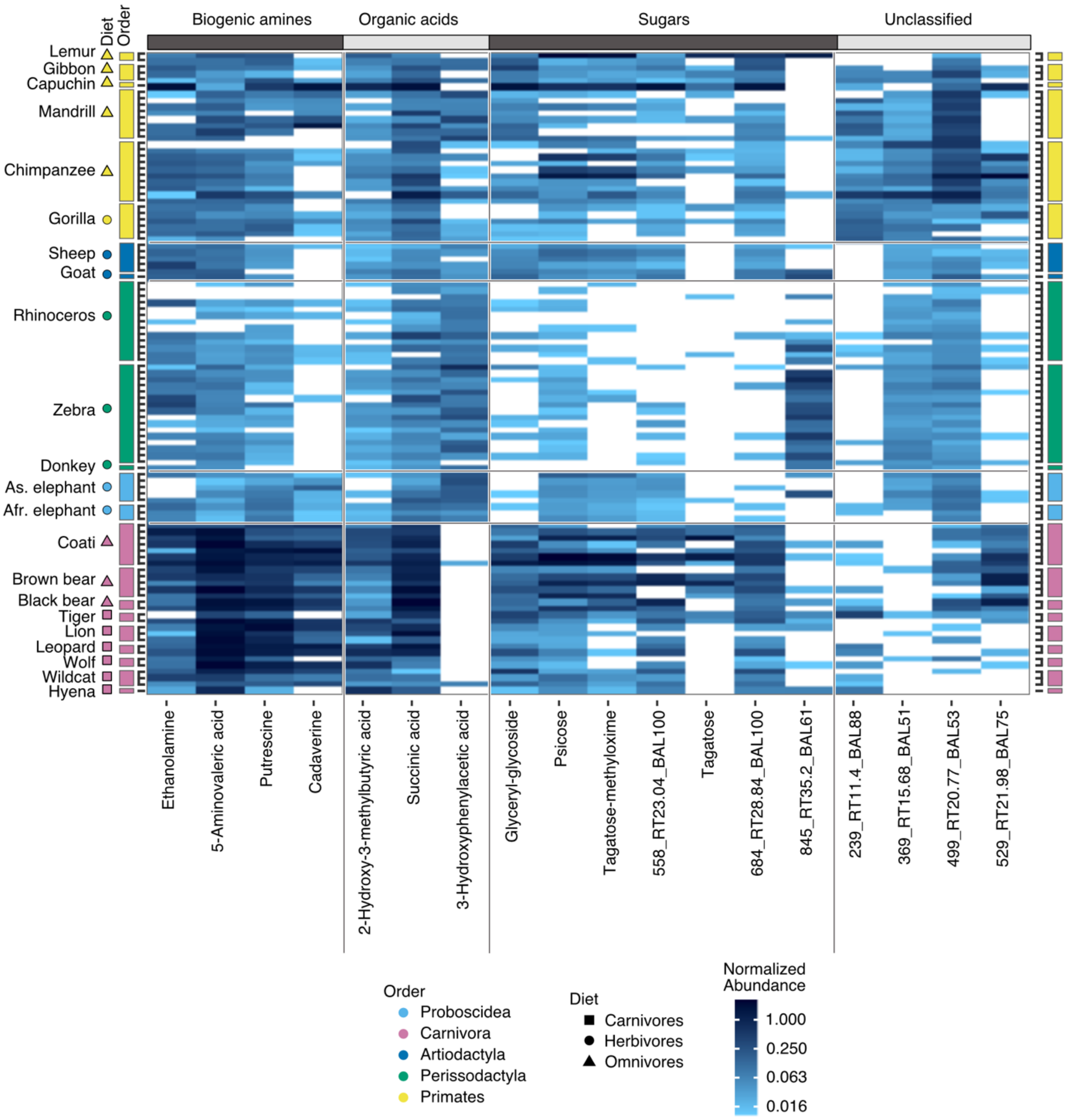

Fig. S14. Heatmap of differential peak features in GC-MS analysis. Unidentified metabolites were named based on index number, retention time (RT), and balance score (BAL) (see methods for more details). Some unidentified metabolites were classified as sugars based on the molecular network analysis.

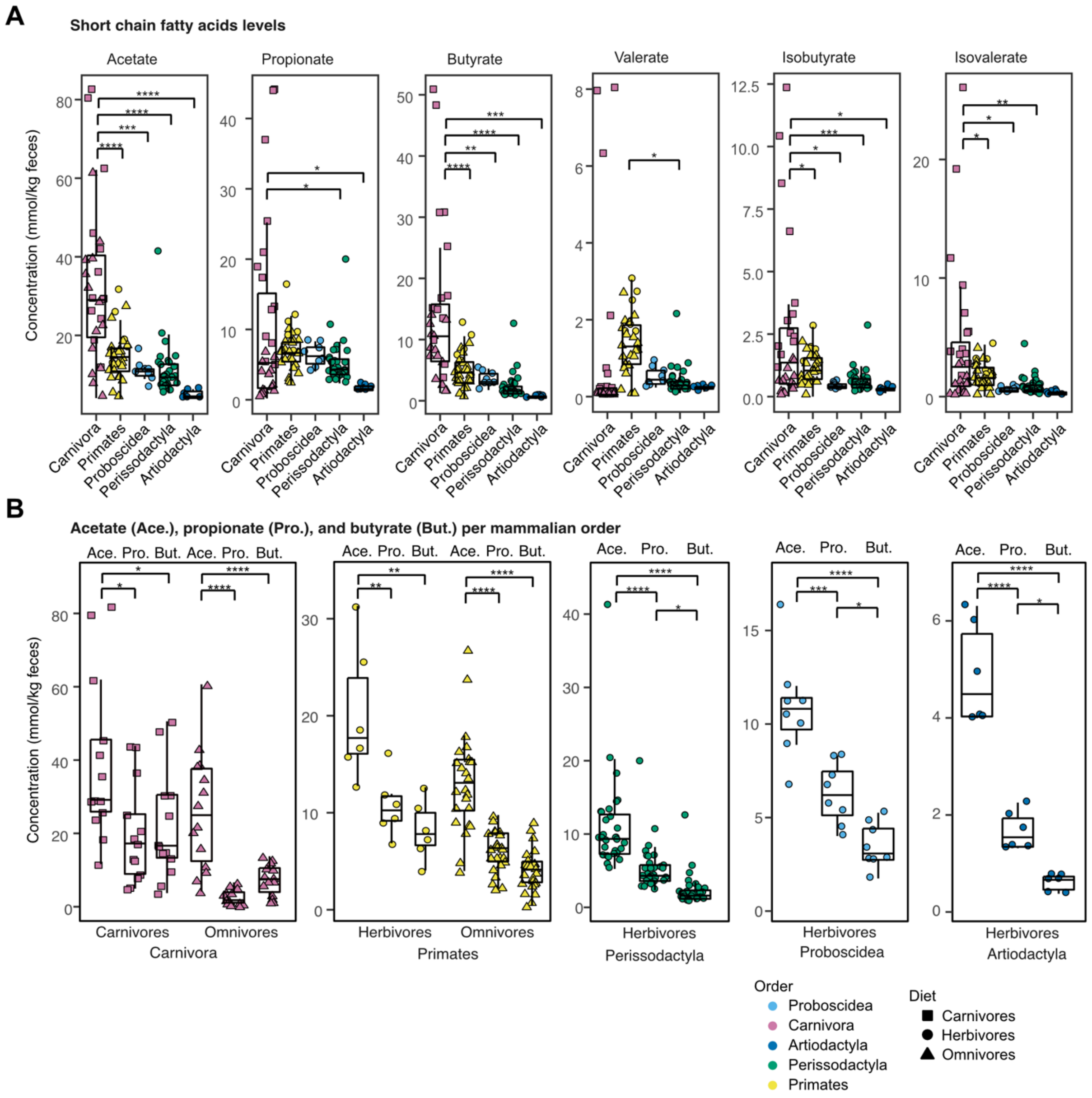

Fig. S15. Short chain fatty acids per mammalian order, compared (A) for each SCFA (A) and (B) per order.

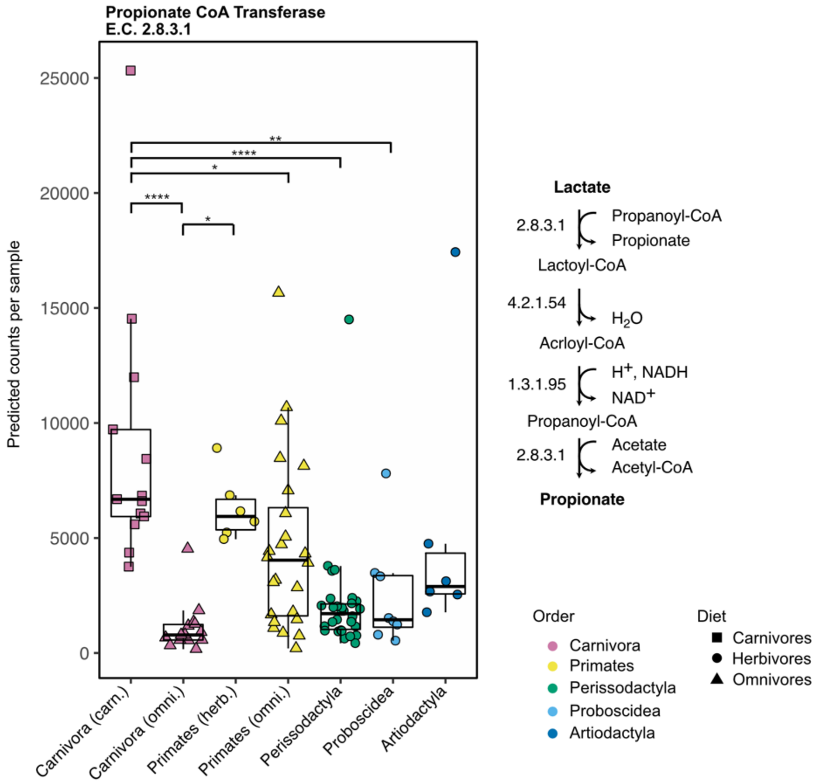

Fig. S16. Omnivores were predicted to contain low levels of propionate CoA transferase (E.C. 2.8.3.1), the first and last enzyme in the acrylate pathway, compared to carnivores and primates (based on PICRUSt2 analysis). All data points are shown, overlaid on a boxplot with the lower and upper hinges corresponding to the first and third quartiles. The significance was determined using one-way ANOVA analysis followed by post-hoc Tukey's test, resulting in adjusted *p* values noted as follows: p<0.05= *, p<0.01= **, p<0.0001= ****.

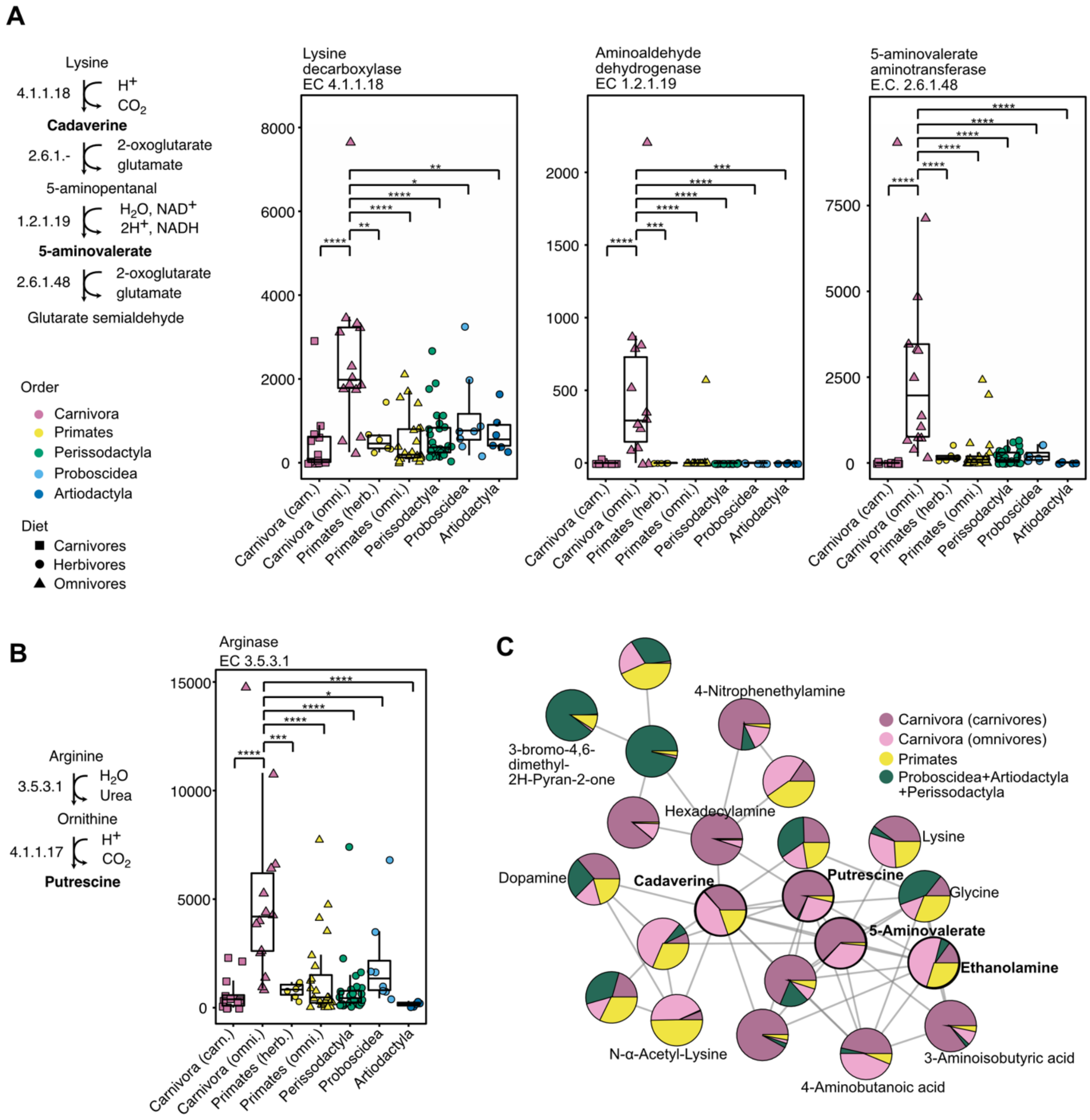

Fig. S17. A. Three key enzymes in the lysine degradation pathway were predicted to be elevated in the omnivorous Carnivora as compared to other mammalian orders (based on PICRUSt2 analysis). B. In the arginine degradation pathway, one enzyme was predicted to be elevated in omnivorous Carnivora. C. Molecular network of biogenic amines. Nodes are colored based on the relative abundance of compounds in each of the four groups. The biogenic amines discussed here are closely linked in the network due to their spectral similarity, and are enriched primarily in Carnivora. (Differentially enriched metabolites are marked in bold). For all panels, all data points are shown, overlaid on a boxplot with the lower and upper hinges corresponding to the first and third quartiles. The significance was determined using one-way ANOVA analysis followed by post-hoc Tukey's test, resulting in adjusted *p* values noted as follows: $p<0.05$= *, $p<0.01$= **, $p<0.001$= ***, $p<0.0001$= ****.

Supplementary methods

DNA extraction

Prior to DNA extraction, samples were treated with a washing protocol adapted from Jami et al. [27] to separate adherent bacteria from fecal material, in order to reduce bias stemming from the low DNA extraction efficiency of particle-associated bacteria. For each frozen sample in PBS-10% glycerol, 2.5 mL was thawed on ice and centrifuged (10,000 g, 20 min, 4°C). The pellet was dissolved in 8 mL extraction buffer (100 mM Tris-HCl, 10 mM ethylenediaminetetraacetic acid (EDTA), 0.15 M NaCl; pH 8.0) and incubated at 4 °C for 1 h with shaking, to enhance the release of particle-associated bacteria into the supernatant. Then, samples were gently centrifuged (500 g, 15 min, 4°C) to separate bacterial cells from the remaining ruptured fecal residues. The supernatant was removed to a new tube and kept on ice, while the pellet was resuspended in 7 mL extraction buffer, followed by another gentle centrifugation (500 g, 15 min, 4°C) to maximize the harvesting of bacterial cells from the fecal material. The supernatants of the first and second gentle centrifugations were combined, and the bacterial cells pelleted from the extraction buffer by fast centrifugation (10,000 g, 20 min, 4 °C). Lastly, the pellet was resuspended in 1.5 mL TE buffer (10 mM Tris–HCl, 1 mM EDTA, pH 8.0), and the solution divided into two 700 µL aliquots and stored at -20°C until DNA extraction. The DNA extraction was performed as previously described by Stevenson et al. [28], with the following minor modifications. After the addition of sodium acetate and isopropanol, the mixture was incubated at -20°C for at least 2 h to enhance DNA precipitation. The precipitated DNA pellet was washed once by adding 1 mL of 70% ethanol. After a final centrifugation, the remaining liquid was removed by drying the DNA pellet, which was then incubated in 30 µl TE at 55°C, for 10 min. The DNA extracts in TE were kept in -20°C until sequencing.

Sample preparation for LC-MS/MS analysis

In general, samples were extracted and run in a semi-randomized order, ensuring that groups of animals were not analyzed sequentially. As a biological quality control (QC) sample, multiple aliquots of an additional fecal sample were extracted and treated alongside the sample set throughout. The extraction protocol was modified from Melnik et al [34]. An aliquot of approx. 1 mL of fecal

suspensions/digested food samples was defrosted in a sonication bath containing ice for 5 minutes and vortexed. Then, 100 µL of each sample was collected using a wide-bore 200 µL pipette tip and deposited in a polypropylene 96-well plate (for fecal samples, each 100 µL aliquot contains approx. 10 mg of feces). Next, 100 µL of methanol (HPLC grade) was added using a multi-channel pipette. The plates were covered with Parafilm and shaken overnight at 4 °C at 300 rpm. The next morning, the plates were centrifuged at 4000 rpm for 15 min at 10°C, and 130 µL of the supernatant was removed and transferred to a microcentrifuge tube (Eppendorf, Hamburg, Germany). The tubes were centrifuged again at 21,130 x g for 10 min at 10°C to remove fine particles prior to MS analysis. Next, 50 µL of each sample was transferred to a glass LC-MS vial with insert containing 100 µL of 50:50 methanol:water solution and 1.5 µM ampicillin as an injection internal standard (final concentration of ampicillin: 1 µM).

Sample preparation for GC-MS analysis

Polar metabolites were extracted in a two-phase extraction modified from a protocol by Giavalisco et al [35]. Two extraction solvent solutions were prepared by weight and precooled to -20 °C: extraction solvent A, composed of 1:3 methyl-*tert*-butyl ether:methanol (MTBE:methanol), and extraction solvent B, composed of 2:3 water:methanol solution with 1.7 µg/mL ribitol (internal standard). First, 300 µL of defrosted fecal samples were deposited in microcentrifuge tubes (Eppendorf) using wide-bore 200 µL pipette tips, 1 mL of extraction solvent A was added, samples were shaken at 1400 rpm at 4 °C for 10 minutes, and then samples underwent sonication in a bath sonicator for 10 minutes. Next, 200 µL of extraction solvent B was added, the samples vortex-mixed, and the samples centrifuged at 21,130 x g at 4 °C for 5 min. The upper organic phase was removed, and then 150 µL aliquots of the lower aqueous-methanol phase were transferred to fresh microcentrifuge tubes. The solvent was removed under reduced pressure at room temperature using a SpeedVac vacuum concentrator, and the dry samples were stored at -20 °C until analysis. Immediately prior to analysis, a two-step derivatization process (methoximation and trimethylsilylation) was carried out according to the protocol described by Lisec et al [36].

Short chain fatty acid (SCFA) extraction

The SCFA extraction was modified from Shabat et al [38]. First, 1 mL aliquots of fecal samples were centrifuged at 21,130 x g at 10 °C for 10 min, and 475 µL of supernatant (fecal water) was transferred to fresh microcentrifuge tubes containing 25 µL of 10 mM 2-ethylbutyric acid in water (internal standard). Samples were frozen at -20 °C until extraction. Then, 50 µL of 25% metaphosphoric acid was added, and samples were vortex-mixed for 2 minutes, then centrifuged at 21,130 x g at 10 °C for 10 min. Next, 400 µL of the supernatant was transferred to a fresh microcentrifuge tube, 400 µL of HPLC-grade methyl-*tert*-butyl ether (MTBE) was added, and the samples were vortex-mixed for 2 minutes then centrifuged at 21,130 x g at 10 °C for 3 min. Lastly, 100 µL aliquots of the supernatant were transferred to glass GC vials containing inserts, and the vials were frozen at -80 °C until injection.

LC-MS/MS analysis

The LC-MS method was modified from Melnik et al [34]. Samples were transferred to the autosampler and stored at 10°C. The injection volume was 5 µL. Samples were chromatographically separated using an Acquity UPLC I-Class System (Waters Corporation), equipped with a HSS T3 chromatography column (1.8 µM, 2.1 x 100 mm; Waters Corporation) heated to 40 °C, and with a BEH C18 Vanguard pre-column (1.7 µM, 2.1 x 5 mm; Waters Corporation). The flow rate was set to 0.4 mL/min, and the composition of the mobile phases were as follows: mobile phase A, 99.9% UPLC-MS grade water+0.1% formic acid; mobile phase B, 99.9% UPLC-MS grade acetonitrile+0.1% formic acid. The following gradient was applied: Initial 1% B, 0-1 min 5% B, 1-8 min 99% B, 8-10.9 min held at 99% B, 10.9-11 min 95% B, 11-13 min 1% B, 13-15 min held at 1% B. MS analysis was performed on an Orbitrap (QExactive, Thermo Fisher Scientific) mass spectrometer equipped with an HESI-II probe and controlled by Xcalibur 3.0 software. The following probe settings were used: capillary voltage of 3500 V, sheath gas (N_2) flow of 60 psi, auxiliary gas pressure (N_2) of 20 psi, capillary temperature of 275°C, and drying gas temperature of 300°C. An external calibration with Pierce LTQ Velos ESI positive ion calibration solution (Thermo Fisher Scientific) was performed prior to data acquisition with error rate less than 1 ppm. A data-dependent MS/MS acquisition mode was used, with a scan range of 100-1500 m/z, and the first 0.5 min

diverted to waste. Full scan at MS1 level was performed with resolution of 70K (profile mode, AGC target 3e6, max. IT 100 ms). The top 5 most intense ions in each MS1 scan were isolated within a 2.0 m/z isolation window (no isolation offset) and fragmented using normalized collision induced dissociation with 30eV. MS2 scans were performed at 17.5K resolution (profile mode, 1 microscan, AGC target 1e5, max IT 60 ms, min. AGC target 8e3, intensity threshold 1.3e5). The MS2 dynamic exclusion parameter was set to 5.0 s.

GC-MS analysis

The samples were injected using the Flex robotic sampling platform (EST Analytical), using an injection volume of 1 µL. Instrument settings for analysis were modified from Hochberg et al [37]. The injected samples were chromatographically separated using a DB-5ms 30 m x 250 µm x 0.25 µm film capillary column on a GC-7820A instrument coupled to a MSD-5977B single-quadrupole mass spectrometer (Agilent Technologies), with helium carrier gas set to a flow rate of 0.723 mL/min and initial oven temperature of 100 °C (equilibriated 3 min pre-run). The inlet was set to split mode with a split ratio of 25:1 (split flow 18.093 mL/min), with the inlet temperature set to 250 °C and the pressure set to 6.7586 psi. The oven temperature was held constant at the initial temperature for 1 min, increased at 1 °C/min to 105 °C (hold time 0 min), and then increased at 6 °C/min to 325 °C with a final hold time of 5 min, and a total run time of 47.667 min. The MSD transfer line was heated to 280 °C, the MS ion source to 230 °C, and the MS quadrupole to 150 °C. The MS acquisition settings were as follows: threshold 150, A/D samples 8, gain factor 1, solvent delay 3 min. Three scan ranges were used sequentially: at start time 3 min, scan range 35-300 m/z; at start time 11 min, scan range 50-450 m/z; start time 19 min, scan range 60-550 m/z.

GC-FID analysis of SCFA.

The SCFA analysis platform was modified from Shabat et al [38]. Samples were analyzed using an Agilent 7890B GC system (Agilent Technologies) coupled to a flame ionization detector (FID). Aliquots (1 µl) were injected with a split ratio of 25:1 into a 30 m x 0.32 mm x 0.25 µm ZEBRON ZB-FFAP column

(Phenomenex) with helium carrier gas set to a flow rate of 2.4 mL/min and initial oven temperature of 100 °C. The oven temperature was held constant at the initial temperature for 5 min, and thereafter increased at 10 °C/min to a final temperature of 125 °C, and a total run time of 12.5 min. The temperatures at the inlet and detector were 250 °C and 300 °C, respectively. Additionally, a calibration curve was prepared in MTBE for a mixture of the following SCFA: acetate, propionate, butyrate, valerate, isovalerate, isobutyrate, and 2-ethylbutyric acid (internal standard). Nine concentration points were prepared ranging from 5 mM to 10 μM. Results were analyzed using the Clarity Chromatography Station (DataApex).

LC-MS/MS MZmine2 workflow

The LC-MS/MS data files were converted to open format mzXML files using the GNPS batch converter, and then processed with MZmine2 v2.34 [39] based on the feature-based molecular networking workflow tutorial (batch converter and tutorial are available at: https://ccms-ucsd.github.io/GNPSDocumentation). The MZmine2 workflow was as follows: (1) Mass detection, MS level 1, centroid mode, noise level 1.0E5, mass list name “masses”; (2) Mass detection, MS level 2, centroid mode, noise level 5.0E2, mass list name “masses”; (3) Chromatogram builder, MS level 1, mass list name “masses”, min time span 0.01 min, min height 3.0E5, m/z tolerance 20 ppm; (4) Chromatogram deconvolution, algorithm wavelets (ADAP), m/z range for MS2 scan pairing: 0.025 Da, RT range for MS2 scan pairing: 0.15 min; (5) Isotopic peaks grouper, m/z tolerance 0.02 m/z, RT tolerance 0.4 min absolute, maximum charge 1, representative isotope most intense; (6) Order peak lists; (7) Join aligner, m/z tolerance 0.02 m/z, weight for m/z: 75, RT tolerance 0.4 min absolute, weight for RT: 25, (8) Peak finder, intensity tolerance 5%, m/z tolerance 0.02 m/z, RT tolerance 0.05 min absolute, RT correction on; (9) Export to CSV file, export row ID, row m/z, row RT, and peak area; (10) Peak list rows filter, Keep only peaks with MS2 scan (GNPS) on, reset the peak ID on; (11) Export for GNPS (mgf file); (12) Export to CSV file, export row ID, row m/z, row RT, row number of detected peaks, and peak area. The last two files were uploaded to GNPS for molecular networking analysis. The CSV file from step 12 was additionally imported into R for subsequent statistical analysis. Peak areas were normalized based dividing by the injection internal standard ampicillin, and log transformed.

GNPS LC-MS/MS parameters

Note: The following description of the parameters is auto-generated by the GNPS platform. The data was filtered by removing all MS/MS fragment ions within +/- 17 Da of the precursor m/z. MS/MS spectra were window filtered by choosing only the top 6 fragment ions in the +/- 50Da window throughout the spectrum. The precursor ion mass tolerance was set to 0.05 Da and a MS/MS fragment ion tolerance of 0.05 Da. A network was then created where edges were filtered to have a cosine score above 0.60 and more than 6 matched peaks. Further, edges between two nodes were kept in the network if and only if each of the nodes appeared in each other's respective top 10 most similar nodes. Finally, the maximum size of a molecular family was set to 100, and the lowest scoring edges were removed from molecular families until the molecular family size was below this threshold. The spectra in the network were then searched against GNPS' spectral libraries. The library spectra were filtered in the same manner as the input data. All matches kept between network spectra and library spectra were required to have a score above 0.7 and at least 6 matched peaks.
